# BYHW Decoction Improves Cognitive Impairments in Rats with Cerebral Microinfarcts via Activation of the PKA/CREB Pathway

**DOI:** 10.1155/2022/4455654

**Published:** 2022-12-30

**Authors:** Bingjie Xue, Bo Ma, Yaoyao Yao, Aimei Zhao, Ying Gao, Jianxun Liu

**Affiliations:** ^1^Institute of Basic Medical Sciences, Xiyuan Hospital, China Academy of Chinese Medical Sciences, Beijing, China; ^2^Institute for Brain Disorders, Beijing University of Chinese Medicine, Beijing, China; ^3^Dongzhimen Hospital, Beijing University of Chinese Medicine, Beijing, China; ^4^Chinese Medicine Key Research Room of Encephalopathy Syndrome and Treatment of the State Administration of TCM, Beijing, China; ^5^Institute of Materia Medica Chinese Academy of Medical Science & Peking Union Medical College, Beijing, China; ^6^Department of Neurology, Dongzhimen Hospital, Beijing University of Chinese Medicine, Beijing, China; ^7^NICM Health Research Institute, Western Sydney University, Locked Bag 1797, Penrith, NSW 2751, Australia

## Abstract

Cerebral microinfarcts (CMIs) are characterized by sporadic obstruction of small vessels leading to neurons death. They are associated with increased risk of cognitive impairments and may have different risk factors compared with macroinfarcts. CMIs have a high incidence and result in heavy social burden; thus, it is essential to provide reasonable treatment in clinical practice. However, there are relatively few researches on the mechanism and treatment of CMIs, and the literature is composed almost exclusively of community—or hospital based on autopsy or imageological studies focusing on elderly patients. The Bu Yang Huan Wu (BYHW) decoction, a traditional Chinese herbal formula, has long been used to treat stroke and stroke-related diseases, including cognitive impairments. We applied microsphere-induced CMI model in rats to investigate the behavioral and molecular consequences of CMIs and to determine how they were ameliorated by BYHW decoction treatment. We then used the Morris water maze, quantitative proteomics, immunohistochemistry, and other molecular assays and found that activation of the PKA/CREB pathway by BYHW decoction treatment may reverse mitochondrial dysfunction, inhibit apoptosis of hippocampal neurons, and ameliorate CMI-induced cognitive impairments in rats. Collectively, these findings confirmed the therapeutic potential of the BYHW decoction in treating cognitive impairments induced by CMIs and demonstrated a viable mechanism for its action.

## 1. Introduction

Cerebral microinfarcts (CMIs) are small ischemic lesions visible on neuropathological examination and detectable in vivo with high-field strength magnetic resonance imaging [1, 2]. CMIs are common in patients with vascular dementia (weighted average 62%), Alzheimer's disease (43%), and demented patients with both Alzheimer-type and cerebrovascular pathology (33%) [3]. CMIs are described as minute foci with neuronal loss, gliosis, pallor, or more cystic lesions. Recent studies have suggested that the presence of CMIs slows down cognitive recovery after acute ischemic stroke [4].

Microvascular obstructed by CMIs leads to ischemia of cerebral neurons. Early evidence has suggested that impaired delivery of oxygen and glucose in response to an ischemic insult can lead to bioenergetic failure of the electron transport chain (driven by mitochondrial complexes) [5, 6], adenosine triphosphate (ATP) depletion, reactive oxygen species (ROS) generation, mitochondrial injury, and apoptosis [7, 8]. The dysfunction of mitochondrial complexes was shown to be a fundamental pathophysiological basis of ischemic injury, subsequently inducing mitochondrial injury and apoptosis [9, 10]. Therefore, maintaining the activity of mitochondrial complexes is critical in attenuating postischemic injury.

Cyclic AMP response element-binding protein (CREB) is a transcription factor activated by protein kinase A (PKA), which regulates a variety of intracellular signaling events, including cellular growth, proliferation, and apoptosis [11–14]. There is increasing evidence to support that except for in the nucleus, CREB exists in the mitochondrial matrix of neurons, where it modulates mitochondrial gene expression and promotes neuronal survival [15, 16]. CREB has been reported to regulate the activity of mitochondrial complexes, especially complex I, and inhibit excessive ROS generation to prevent stress-induced mitochondrial apoptosis [17, 18]. However, we and other groups demonstrated that ischemic insults led to the activity of PKA and CREB inhibited along with mitochondrial damage [18, 19]. Such findings linking the PKA/CREB pathway to mitochondrial regulation have engendered new perspectives to treatment strategies for neurological diseases [20, 21].

The Bu Yang Huan Wu (BYHW) decoction has been widely used in China to treat stroke and stroke-related diseases since the 17^th^ century. It is one of the most frequently prescribed medications for ischemic or hemorrhagic stroke in China [22–25]. One study reported that BYHW decoction, in combination with cholinesterase inhibitor donepezil, which is a medicine used to treat Alzheimer's disease, improved cognitive function in stroke patients in the clinic [26]. A matched study in Taiwan found that stroke patients who received BYHW decoction therapy had a lower long-term risk of epilepsy [24]. Other pharmacodynamic properties of this decoction show positive effects in chronic glomerulonephritis and poststroke epilepsy [27, 28]. Despite such success in humans for brain protection, the molecular underpinnings of the therapeutic action of this herbal treatment have not been clearly elucidated and we aimed to investigate this mechanistic question. The BYHW decoction is composed of *Astragalus membranaceus* Fisch. (Huang Qi), *Ligusticum sinense* Oliv. (Chuan Xiong), *Angelica sinensis* (Oliv.) Diels (Dang Gui), *Carthamus tinctorius* L. (Honghua), *Paeonia lactiflora* Pall. (chi shao), *Prunus persica* (L.) Stokes (Tao Ren), and *Lumbricus* Linnaeus (Di Long). Numerous studies have reported that the bioactive phytocompounds in these plants (including Formononetin, Astragaloside IV, Ferulic acid, Paeoniflorin, Benzoylpaeoniflorin, Senkyunolide I, Senkyunolide H, Hydroxysafflor yellow A, and Amygdalin) could ameliorate the consequences of an ischemic insult by anti-inflammatory, antioxidant, antiapoptosis, and immunomodulatory effects to protect the integrity of the blood-brain barrier, retain neuronal morphology, and ameliorate learning and memory impairments [29–36]. We discovered previously that Astragaloside IV, the main bioactive phytocompound in BYHW decoction, protects primary cerebral cortical neurons from oxygen and glucose deprivation/reoxygenation by activating the PKA/CREB pathway. Therefore, in this study, we hypothesized that the BYHW decoction activates the PKA/CREB pathway to reverse mitochondrial dysfunction and inhibit apoptosis, thereby ameliorating microinfarction-induced cognitive impairments. To test this hypothesis, we used microsphere-induced CMI model and conducted behavioral, molecular, and bioinformatics assays and analyses.

## 2. Materials and Methods

### 2.1. Chemical and Reagents

#### 2.1.1. Animal Assays

Materials used for animal assays include the following: mitochondria isolation kit (Sigma, MITOISO1); in situ cell death detection kit (Roche, 11684817910); fatty acid-free albumin bovine V (BSA) (Merck, 126575); HEPES (Sigma, H4034); mannitol (Sigma, 78513); sucrose (Sigma, v900116); EGTA (Sigma, E3889); rotenone (Sigma, R8875); succinate (Sigma, S2378); ADP (Sigma, a2754); oligomycin (Aladdin, 1404-19-9); KH_2_PO_4_ (Sigma, 1551139); MgCl_2_ (Sigma, 8266); malic acid (Sigma, M6413); sodium pyruvate (Sigma, P5820); FCCP (Sigma, C2920); antimycin A (Sigma, A8674), ascorbic acid (Sigma, A5960); N,N,N′,N′-tetramethyl-p-phenylenediamine dihydrochloride (TMPD) (Sigma, t106596); PKA (Abcam, ab216572); p-PKA (Abcam, ab75991); CREB (Abcam, ab32515); p-CREB (Abcam, ab32096); Actin (Abcam, ab6276); Tomm20 (Cell Signaling Technology, 42406); ATPlite luminescence ATP detection assay system (PerkinElmer, 6016941); cAMP (Abcam, ab133051); JC-1 dye (Life technologies, T3168); H2DCFDA (Life technologies, C6827); MitoTracker red (Life technologies, M7512); MitoTracker green (Life technologies, USA, M7514); MitoSOX Red (Life Technologies, M36008); Annexin V-FITC/PI (Solarbio, China, CA1020); ATP content kit (Solarbio, China, BC0305); and reference substances of Formononetin, Astragaloside IV, Ferulic acid, Paeoniflorin, Benzoylpaeoniflorin, Senkyunolide I, Senkyunolide H, Hydroxysafflor yellow A, and Amygdalin bought from the National Institutes for Food and Drug Control (Beijing, China).

#### 2.1.2. Quantitative Proteomics

Reagents used for quantitative proteomics include the following: tris (2-carboxyethyl) phosphine (TCEP) (Sigma, C4706); iodoacetamide (IAM) (Sigma, I1149); and trypsin (Promega, 5111, enzyme/protein ratio of 1 : 25 w/w).

### 2.2. Preparation and Analysis for BYHW Decoction

BYHW decoction was provided by Jining Huaneng Co., Ltd. (Jining, China, 200808), which was composed of *Astragalus membranaceus* Fisch., 84.2 g/100 g; *Ligusticum sinense* Oliv., 2.1 g/100 g; *Angelica sinensis* (Oliv.) Diels, 4.2 g/100 g; *Carthamus tinctorius* L., *2.1* g*/100* g; *Paeonia lactiflora* Pall., 3.2 g/100 g; *Prunus persica* (L.) Stokes, 2.1 g/100 g; and *Lumbricus* Linnaeus, 2.1 g/100 g. The main compounds of BYHW decoction were identified according to the literature [29–36]. Reversed-phase nanocapillary ultra-high-pressure liquid chromatography coupled to tandem mass spectrometry (UPLC-MS/MS) was used to obtain the main ingredients of BYHW decoction. Firstly, samples of BYHW decoction were separated on Waters ACQUITY UPLC T3 (2.1 × 100 mm column, 1.8 *μ*m); mobile phases consisted of solvents A (acetonitrile) and B (pure water), as shown in Supplementary Table 1A. Then, the ingredients were detected by MS, and the parameters were shown in Supplementary Table 1B.

### 2.3. Animals

Adult, male Sprague-Dawley (SD) rats (200–220 g) were supplied by the Experimental Animal Center of Xiyuan Hospital (Beijing, China, License number: SCXK (Beijing) 2016-0006). Animals were housed under standard laboratory conditions with free access to animal chow and water. All experimental procedures were carried out according to the protocols approved by the Ethics Committee for Animal Experimentation of Xiyuan Hospital and in accordance with the National Institutes of Health Guide for the Care and Use of Laboratory Animals. All rats were given at least 48 h to acclimatize before any surgeries or experiments were conducted.

### 2.4. CMI Model

**Figure 1:**
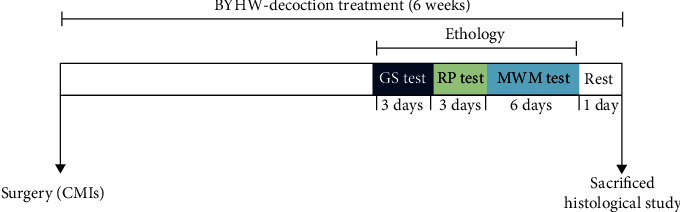
Timeline of the CMI model, BYHW decoction treatment, and behavioral testing. GS: grip strength; RP: rotating pole; MWM: Morris water maze.

The timeline of the CMI model and BYHW decoction treatment is illustrated in Figure 1.

CMI surgery was performed as previously described with the modifications specified here [37–40]. Firstly, the right common carotid artery (CCA) for the rats was temporarily occluded with vascular clamps, and pterygopalatine artery (PA) was occluded by electrocoagulation. Next, the external carotid artery (ECA) was tied off and cut, and the syringe containing the microspheres (0.2 mL) was then inserted into the previously cut ECA and tied in place with suture. Briefly, serum was withdrawn from a donor rat and mixed with microspheres (12 *μ*m, UVPMS-BY, Sigma, USA) for a final microsphere concentration of 1 mg/mL. Male SD rats weighing 200–220 g were anesthetized with isoflurane, and their body temperatures were maintained around 37°C. A cervical midline incision was made to expose CCA and its branches: the internal carotid artery (ICA), ECA, and PA. Then, a 0.4-millimeter stenosis was created on the CCA proximal to the carotid bifurcation. Next, the ECA was tied off and cut, and the PA was clamped with a 10 mm microaneurysm clamp. The syringe containing the microspheres (0.2 mL) was then inserted into the previously cut ECA and tied in place with suture. Finally, the ICA, CCA, and PA clamps, as well as the CCA stenosis were removed, and the incision was closed.

### 2.5. BYHW Decoction Treatment

Sixty rats subjected to CMIs were divided into four equal groups (15 rats per group) and administered either saline solution (the Vehicle control), 25.2 mg/kg EGb761 (ginkgo biloba extract) in saline (the positive control), 3.78 g/kg BYHW decoction in saline, or 7.56 g/kg BYHW decoction in saline by oral gavage. In addition, fifteen Sham-operated rats were treated with saline solution. Administration of the above solutions was repeated once every day at the same time for six weeks. The timeline of the treatment paradigm is illustrated in Figure 1.

### 2.6. Morris Water Maze

The Morris water maze task was conducted as described previously [41]. Briefly, the rats were trained to perform the task for five consecutive days, and then, the probe trial was conducted on day 6. The rats were allowed to enter the pool of water from four locations (N, S, E, and W), and the order of these entry points was changed daily in a random manner. The rats were trained four times per day, during which they swam until they found the hidden platform or were guided to it by the experimenter if not found within 120 s. They were allowed to stay on the platform for 30 s before the next swim trial. The time taken to find the platform was measured as the latency time, which determined the spatial learning ability of the rats. Single probe trials (where the hidden platform was removed) were conducted on the day after the last training session and used to evaluate spatial memory ability. In the probe trial, the rats were released from a random start position and allowed to swim for 120 s in the absence of the platform. Their tracks were recorded using a video camera and the EthoVision software (Noldus), and the number of target annulus crossovers was compared.

### 2.7. Motor Impairments

Psychomotor coordination and limb dystonia were assessed using the accelerating rotarod [42] and forelimb strength [43]. In brief, an elevated wooden pole (size 600 mm) was rotated at 10, then 20, and then 40 rotations per minute (rpm). Rats were trained to keep running on the pole at the appropriate speed to avoid falling on the floor. Every animal was trained on the rotarod spinning once a day for 3 days before the CMI surgery, and the acceleration was from 10 to 40 rpm over a 5-minute period. Then, animals were tested on the fifth week after CMIs at 40 rpm per minute. The rats' performance was evaluated based on the length of time they stayed on the pole.

To analyze limb dystonia, forelimb strength was measured using the Grip Strength Test Meter GS3. Rats were allowed to voluntarily grip a grid and pull backward with either the healthy or the paralyzed forelimb. The maximum force of their pull was recorded, and the average measurement out of three trials was taken for analysis.

### 2.8. Immunohistochemistry, TUNEL (Terminal Deoxynucleotidyl Transferase dUTP Nick End Labeling), and HE Staining (Hematoxylin and Eosin)

Immunohistochemical staining was performed using the standard avidin-biotin complex peroxidase method on formalin-fixed, paraffin-embedded brain tissue sections. To determine PKA and CREB expressions and activity, sections were incubated with 0.3% H_2_O_2_ in methanol for 15 minutes and then submerged in blocking solution (1% albumin bovine in PBS) for 1 hour at room temperature prior to staining. Primary antibodies, such as PKA (1 : 500), p-PKA (1 : 2500), CREB (1 : 500), and p-CREB (1 : 100) were applied on sections with overnight incubation at 4°C. After three washes with PBS, sections were incubated with horseradish peroxidase-conjugated secondary antibodies for 0.5 hours at 37°C. The mean optical density was quantified with the Image-Pro Plus software (Image-Pro Plus 6.0) (10× magnification) in a blinded manner.

TUNEL was used to determine the rate of neuronal apoptosis in the hippocampus according to the manufacturer's instructions. Eight brain sections from each animal were selected for TUNEL staining, and the number of TUNEL-positive cells was counted. Image acquisition and cell counting were conducted with the Image-Pro Plus software (Image-Pro Plus 6.0) (10× magnification) in a blinded manner.

HE staining was done to evaluate neuronal features and count cell numbers. The paraffin sections were subjected to dewaxing and hydration, followed by hematoxylin staining for 5 min. The slices were then differentiated with 0.6% hydrochloric acid alcohol for 30 s, counterstained in acidified eosin alcohol (pH 4.2) for 3 min, and dehydrated and cleared. Images were captured and cell number was determined using computer-imaging software (Image-Pro Plus 6.0) (10× magnification) in a blinded manner.

### 2.9. Transmission Electron Microscopy

Rat hippocampi from different treatment groups were washed with PBS and then fixed with 2.5% glutaraldehyde in 0.1 M sodium cacodylate buffer for 2 h. Following three washes with 0.1 M sodium cacodylate buffer, the hippocampi were further fixed with 1% osmium tetroxide in 0.1 M sodium cacodylate buffer for 1.5 h. They were then dehydrated in an ascending series of alcohol concentrations and embedded in epoxy resin. Ultrathin sections (50 nm) of the hippocampi were sliced and stained with 4% uranium acetate for 30 min followed by Sato's lead staining solution. Samples were examined using a Hitachi transmission electron microscope.

### 2.10. Isolation of Mitochondria

Mitochondria were isolated from the rat hippocampi following the manufacturer's protocol. Briefly, the fresh brain hippocampi were homogenized with 10 volumes of 1× extraction buffer (10 mM HEPES, 200 mM mannitol, 70 mM sucrose, and 1 mM EGTA; pH 7.5) containing 2 mg/mL albumin on ice. The homogenate was centrifuged at 800 × *g* for 5 min and the supernatant was collected for further centrifugation at 3,500 × *g* for 10 min. After removing the supernatant, the pellets were again resuspended in 10 volumes of extraction buffer and the centrifugation steps (800 × *g* for 5 min, supernatant collected, and 3,500 × *g* for 10 min) were repeated. The resulting pellet was suspended in 1× MSHE+BSA (210 mM mannitol, 70 mM sucrose, 5 mM HEPES, 1 mM EGTA, and 0.5% (*w*/*v*) fatty acid-free BSA; pH 7.2), and its protein concentration was measured using a bicinchoninic acid (BCA) assay [44].

### 2.11. Measurement of Membrane Potential (ΔΨ*m*) from Isolated Mitochondria in the Rat Hippocampi

The mitochondrial membrane potential (ΔΨ*m*) was estimated using the dye JC-1 (5,5′,6,6′-tetrachloro-1, 1′,3,3′-tetraethylbenzimidazole carbocyanine iodide) by flow cytometry. JC-1 is a lipophilic fluorescent cation that exists in the cells/mitochondria as green fluorescent monomers at low ΔΨ*m* or as red fluorescent aggregates at greater ΔΨ*m*. Mitochondria were incubated for 20 min at 37°C after the corresponding treatment in medium containing 10 *μ*M JC-1. The mitochondria were then rinsed twice in PBS and immediately analyzed by flow cytometry. Measurements were carried out on three rats per group, in duplicate for every rat. Data was collected at emissions of 529 nm for green fluorescence and 590 nm for red fluorescence. The red/green fluorescence intensity ratio was calculated to represent the mitochondrial membrane potential.

### 2.12. Measurement of Electron Flow (Mitochondrial Complex I-IV) in the Mitochondria Isolated from the Rat Hippocampi

An XF96 analyzer (Seahorse Bioscience) was used to measure the freshly isolated hippocampal mitochondria (4 *μ*g of mitochondrial protein per well). At first, the isolated mitochondria were incubated in MAS solution (70 mM sucrose, 220 mM mannitol, 10 mM KH_2_PO_4_, 5 mM MgCl_2_, and 2 mM N-2-hydroxyethylpiperazine-N-ethane-sulphonic acid (HEPES), 1 mM ethylene glycol bis-amino tetmacetate (EGTA), and 0.2% (*w*/*v*) fatty acid-free bovine serum albumin (BSA) in the presence of 2 *μ*M malic acid, 10 mM sodium pyruvate, 4 mM adenosine diphosphate (ADP), and 4 *μ*M carbonyl-cyanogen-trifluoromethylphenylhydrazine (FCCP), pH 7.2 at 37°C. Next, the oxygen consumption rate (OCR) of each mitochondrial complex was measured with the sequential addition of 2 *μ*M rotenone, 10 mM succinate, 1.5 *μ*g/mL antimycin A, and a solution of 10 mM ascorbic acid and 0.1 mM N,N,N′,N′-tetramethyl-p-phenylenediamine dihydrochloride (TMPD). Measurements were carried out on three rats per group, in duplicate for every rat. Specifically, variation of OCR before and after injection of rotenone, a complex I inhibitor, revealed the activity of complex I (between 5.8 and 16 min). Further addition of succinate revealed complex II-driven respiration, and antimycin A (AA) determined the activity of complex II/III (between 16 and 26.2 min). Finally, complex IV activity was tested following the addition of ascorbate and TMPD (between 36.5 and 46.6 min) [45].

### 2.13. OCR Measurement of Mitochondrial Complex V from Isolated Rat Mitochondria

Isolated rat hippocampal mitochondria (4 *μ*g/well) were seeded on another Seahorse 96-well plate and diluted in MAS solution with 2 *μ*M rotenone, 10 mM succinate, and 4 mM ADP (a protective agent for the mitochondria that supported basal respiration in the electron flow assay). OCR of complex V was measured following the injection of 22 *μ*L (25 *μ*g/mL) oligomycin (inhibitor of complex V) on the Seahorse XF96 flux analyzer according to Luso et al. [45].

### 2.14. ROS Production from the Isolated Mitochondria in the Rat Hippocampi

To analyze the production of ROS, the isolated mitochondria (20 *μ*g) were suspended in 1 mL of MAS solution containing complex I substrate malic acid with or without 10 *μ*M H2DCFDA. Mitochondria were incubated for 20 min at 37°C, and the fluorescent signal from dichlorofluorescein (DCF; excitation 488 nm and emission 525 nm) was immediately analyzed by flow cytometry.

### 2.15. ATP-Release Assay in the Rat Hippocampi Mitochondria

ATP release was detected using the ATPlite Luminescence ATP Detection Assay System according to the manufacturer's protocol with a multifunction microplate reader. Briefly, the brain hippocampi were homogenized with 10 volumes of 1× lysis buffer from an ATP detection kit on ice. A BCA assay was used to quantify the protein content of each sample (hippocampal tissue homogenate). Next, 50 *μ*L substrate was added to the cell lysates, and ATP content was measured (PerkinElmer EnSpire™, USA) after a 5-minute incubation. The resultant chemiluminescence value was normalized to the protein content of each well.

### 2.16. Mitochondrial Damage, Neuronal Apoptosis, ROS, and ATP Content Measurements In Vitro

Primary hippocampal culture was prepared [46] as previously described. Primary hippocampal neurons cultured in 6-well plates were underwent OGD or not (naïve group) after 5–6 days in culture [19]. To obtain the extract, the BYHW decoction was suspended in HBSS at a concentration of 35 mg/mL, mixed thoroughly with a vortex for 5 minutes, and centrifuged at 150 g for 30 minutes to collect the supernatant. Mitochondria damage, apoptosis, ROS, and ATP content were detected using MitoTracker kit, Annexin V-FITC/PI kit, MitoSOX red kit, and ATP content kit according to the instruction of the manufactures, respectively. Damage to the mitochondria and neuronal apoptosis was measured in a BD FACSAriaTM llu flow cytometer and analyzed using the DIVA software from BD Biosciences (San Jose, CA, USA). ROS in cells were evaluated by fluorescent microplate reader (PerkinElmer EnSpire™, USA). ATP content was measured (PerkinElmer EnSpire™, USA) at 340 nm immediately and after 3-minute incubation at 37°C. All experiments were performed at least three times.

### 2.17. Identification of Protein Expression by Proteomic Analysis and Parallel Reaction Monitoring (PRM) in the Rat Hippocampi

Samples of approximately 25 mg rat hippocampi were placed in sample tubes and homogenized in 1 mL lysis buffer (10 mM HEPES/NaOH, pH 7.4, and 1 mM EDTA containing protease-inhibitor cocktail) by ultrasound for 5 cycles of 20 s on ice. Then, the tissue debris was removed by centrifugation at 15,000 rpm for 20 min at 4°C, and the supernatant was collected. Protein samples were treated with 5 mM of tris(2-carboxyethyl)phosphine (TCEP) and 10 mM of iodoacetamide (IAM) and then digested with trypsin (enzyme/protein ratio of 1 : 25 *w*/*w*) at 37°C rocking overnight. The peptides were analyzed by nanoflow liquid chromatography-tandem mass spectrometry (LC-MS/MS) using a Q-Exactive-plus mass spectrometer. Data-dependent acquisition (DDA) analysis was used for protein identification and quantification by the Proteome Discoverer and MaxQuant software. A filter of 1% false discovery rate (FDR) for protein level was set. Finally, parallel reaction monitoring (PRM) was used to monitor the peptides of selected proteins, and the sum of peak areas for the most intense product ions was considered for quantification, which was conducted with the Skyline software [47].

### 2.18. Measurement of Cyclic AMP (cAMP) Content

Mitochondria isolated from the rat hippocampi were collected using a cAMP complete enzyme-linked immunosorbent assay kit according to the manufacturer's instructions. The following formula was used to calculate cAMP content: cAMP relative content = (1 − *S*/*B*_0_) × 100, where *S*=samples (pmol/mL) and *B*_0_=0 pmol/mL standard.

### 2.19. Statistical Analysis

The data were analyzed by SPSS or GraphPad Prism, and an ANOVA was used. *P* < 0.05 was considered a statistically significant difference. The ANOVA was followed by Tukey's HSD post hoc test if homogeneity of variance was met or by the Games-Howell post hoc test if variance was unequal.

## 3. Results

### 3.1. Quantitative Analysis of Bioactive Compounds in BYHW Decoction

**Figure 2:**
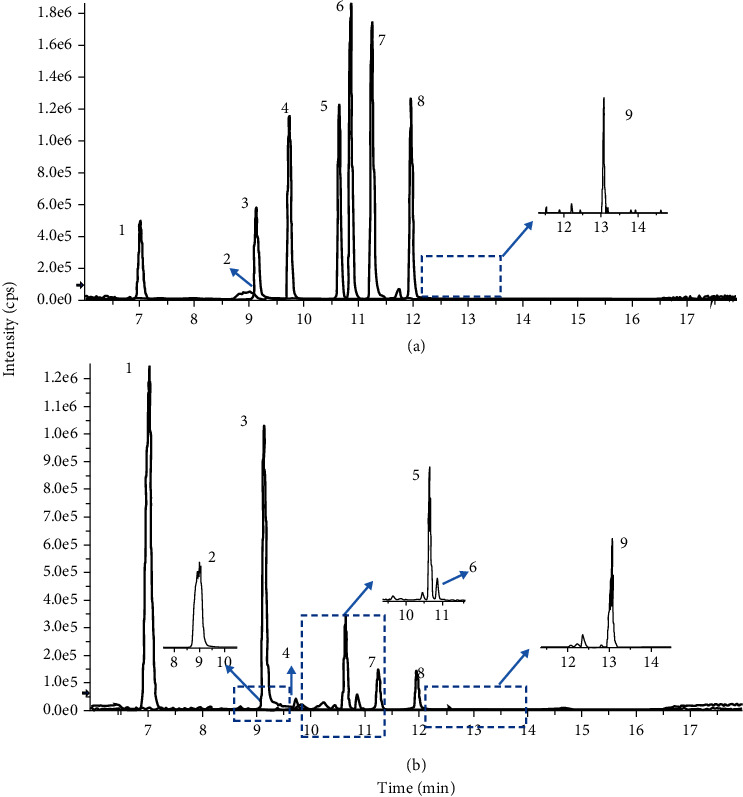
Multiple reaction monitoring (MRM) superposition of mixed standard curves (a) and representative mass spectrum of the primary compounds in BYHW decoction (b): (1) Amygdalin, (2) Hydroxysaffor yellow A, (3) Paeoniflorin, (4) Ferulic acid, (5) Senkyunolide I, (6) Senkyunolide H, (7) Benzoylpaeoniflorin, (8) Formononetin, and (9) Astragaloside IV.

UPLC-MS/MS was used to determine the contents of representative chemical components in BYHW decoction.  Figure 2 shows the chromatograms of the main identified components of BYHW decoction, and the original image of MS1 and MS2 is illustrated in Supplementary Figure 1. In Table 1, the calibration curves' equations and the quantitative limits of these components were determined, and all calibration curves show good linear regression (*R*^2^ > 0.99). The concentration of the main compounds is calculated using the calibration curve of the internal standard in Table 2.

### 3.2. BYHW Decoction Improved CMI-Induced Cognitive and Motor Impairments

**Figure 3:**
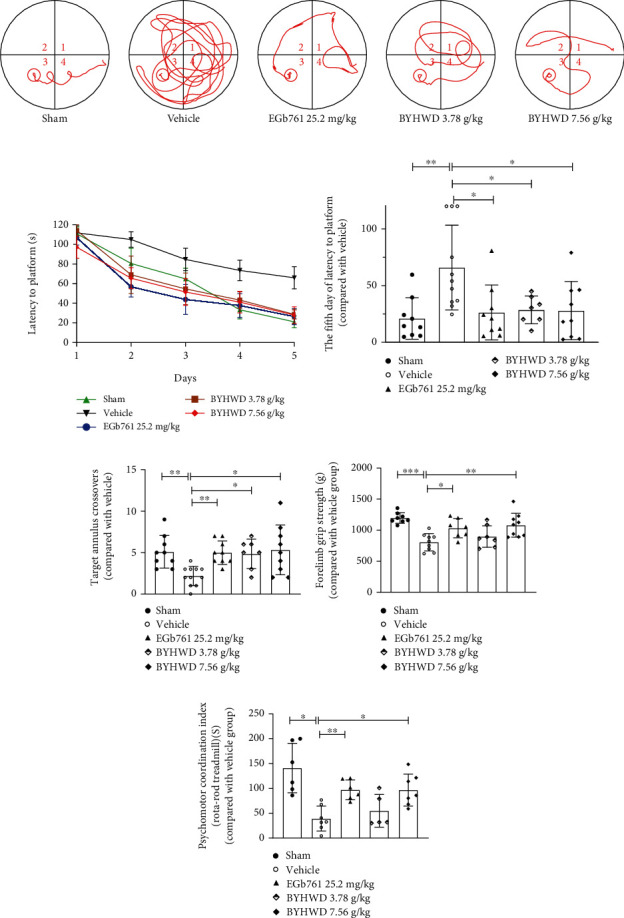
BYHW decoction inhibited CMI-induced cognitive and behavioral impairments. (a) Representative pictures of the swim tracks during the training period (spatial learning phase) of the Morris water maze task. (b) Time taken to reach the hidden platform (latency to platform) on successive days during the training period for the Morris water maze and the quantification of results on the fifth day (*n* = 7‐11 rats). (c) Number of target annulus crossings during the probe trial (*n* = 7‐11 rats). (d, e) BYHW decoction treatment affected the CMI-injured rats' strength and locomotor balance by grip strength (*n* = 7‐9 rats) and rotating pole tests (*n* = 5‐7 rats). The values are expressed as the mean ± SD and analyzed by one-way ANOVA. ^∗^*P* < 0.05, ^∗∗^*P* < 0.01, and ^∗∗∗^*P* < 0.001 versus the Vehicle group.

To investigate whether BYHW decoction treatment can attenuate CMI-related cognitive impairments, we used the Morris water maze to examine cognitive function in the fifth and sixth weeks following the CMI injury. During the training period, rats in the Vehicle group showed consistently longer latencies in locating the hidden platform. They moved more along the wall of the pool and even on the fifth day, which was the last day of training (spatial learning phase); their latency time was 3.14 times that of the Sham group (Figure 3(b)). While rats in all the BYHW decoction treatment groups were quicker to find the platform even on the second day of training, the differences in latency were most evident on day 5. Specifically, both the 3.78 g/kg and 7.56 g/kg BYHW decoction treatments shortened latencies by over 50%, respectively, compared with Vehicle treatment (Figure 3(b)). On the subsequent probe test without the platform (to assess spatial memory), these Vehicle-treated rats failed to show any recall of the target platform location. They spent more time in the periphery of the pool and only crossed the target annulus region with an average of twice. The rats that were administered with the BYHW decoction performed better, with an average of 4–5 annulus crossovers (Figure 3(c)).

To further investigate whether BYHW decoction treatment can attenuate CMI-related behavioral impairments, we used the grip strength and rotating pole tests before the Morris water maze tests. We found that BYHW decoction treatment attenuated the CMI-induced psychomotor incoordination and limb dystonia (Figures 3(d) and 3(e)).

Taken together, these results showed that BYHW decoction treatment effectively attenuated CMI-induced cognitive and behavioral impairments in rats.

### 3.3. BYHW Decoction Attenuated CMI-Induced Neuronal Injury

**Figure 4:**
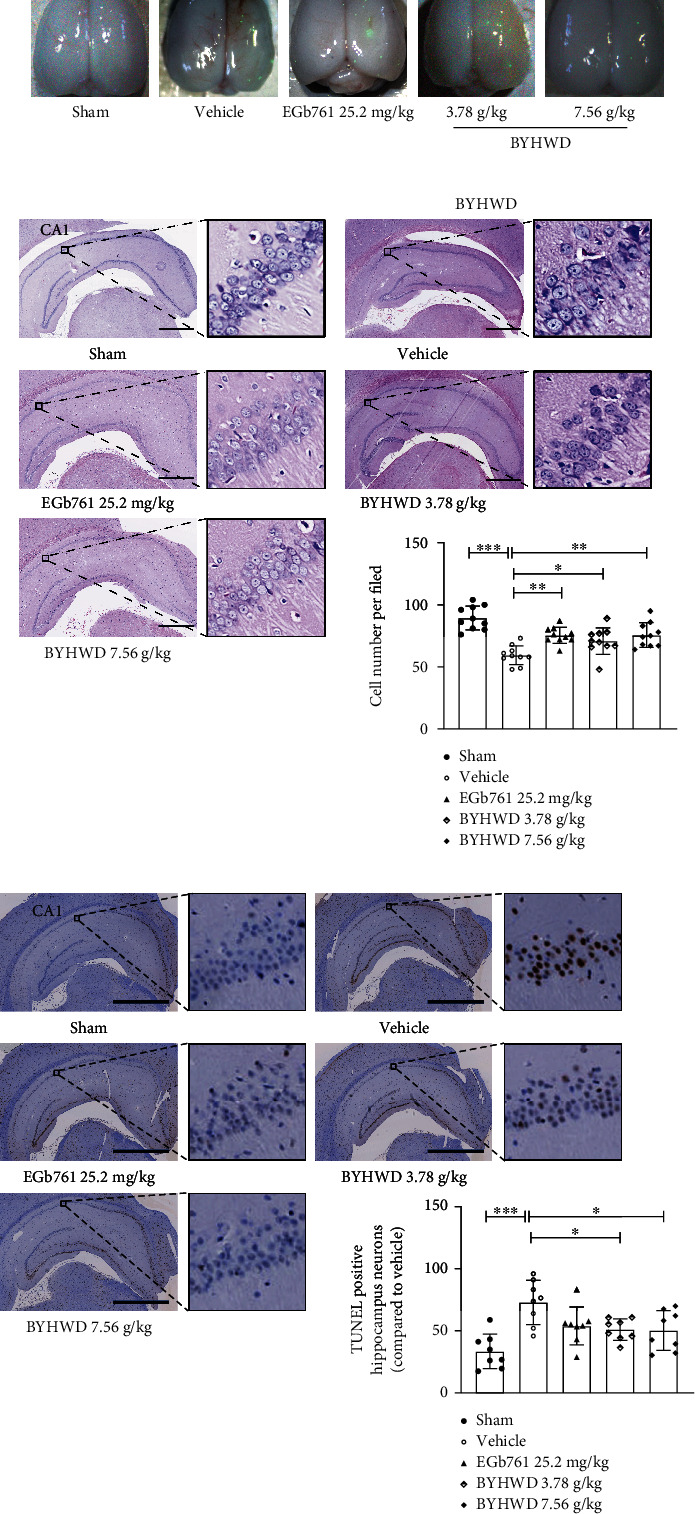
Effects of the BYHW decoction in the CMI model. (a) Representative images of the rat whole brains following the CMI injury and the 6-week administration of 3.78 or 7.56 g/kg BYHW decoction are shown. During the CMI surgery, all groups except for the Sham group were injected with 0.2 mL fluorescent microspheres. The images were obtained under ultraviolet light excitation to visualize the microspheres and ensure that the model was successful. (b) Effects of BYHW decoction on cell morphology and number in the hippocampus. Representative images are shown from the same area of CA1 (scale bar, 1 mm), where cell numbers were counted under 10× magnification. The value for each rat was determined from the average of four different slices (*n* = 3 rats). (c) Representative images of hippocampi with TUNEL staining (scale bar, 1 mm) showing apoptotic cells (brown nuclei). Quantitative analysis of the number of apoptotic neurons in the hippocampus is shown in the bar chart (*n* = 3 rats). Data were analyzed by one-way ANOVA. ^∗^*P* < 0.05, ^∗∗^*P* < 0.01, and ^∗∗∗^*P* < 0.001 versus the Vehicle group.

To determine the underlying mechanisms behind the protective effects of BYHW decoction against CMI-induced cognitive impairments, we studied the changes in cell morphology and number by histological examination following BYHW decoction treatment. As shown in Figure 4(a), the fluorescent points that appeared under ultraviolet light excitation confirmed the entrance of the microspheres into small or microarteries in many different brain regions. HE staining showed that neurons in the Sham group exhibited well-organized architecture with a round-shaped and clear nuclei, while the CMI-injured rat neurons were poorly structured with a pyramidal appearance and shrunken nuclei. CMI injury also led to a significant decrease in the number of cells. After the 6-week treatment paradigm, BYHW decoction was found to abate these pathological features, as demonstrated by the improved neuronal morphology, organization, and number (Figure 4(b)). The TUNEL assay was conducted to examine the neuroprotective effects of the BYHW decoction against ischemia-induced apoptosis in the hippocampus. Rats that were injured by CMIs (Vehicle group) showed a higher proportion of TUNEL-positive neurons compared with those in the Sham group. Hippocampi from animals treated with 3.78 and 7.56 g/kg BYHW decoction showed a reduced proportion of TUNEL-stained apoptotic cells compared with the Vehicle-treated rats (Figure 4(c)). Taken together, our histological results clearly demonstrated the antiapoptotic effect of BYHW decoction on neurons, which helps to explain its neuroprotection benefits against cognitive impairments on the cellular level.

### 3.4. BYHW Decoction Protected Mitochondrial Function and Morphology in CMI-Injured Rats

**Figure 5:**
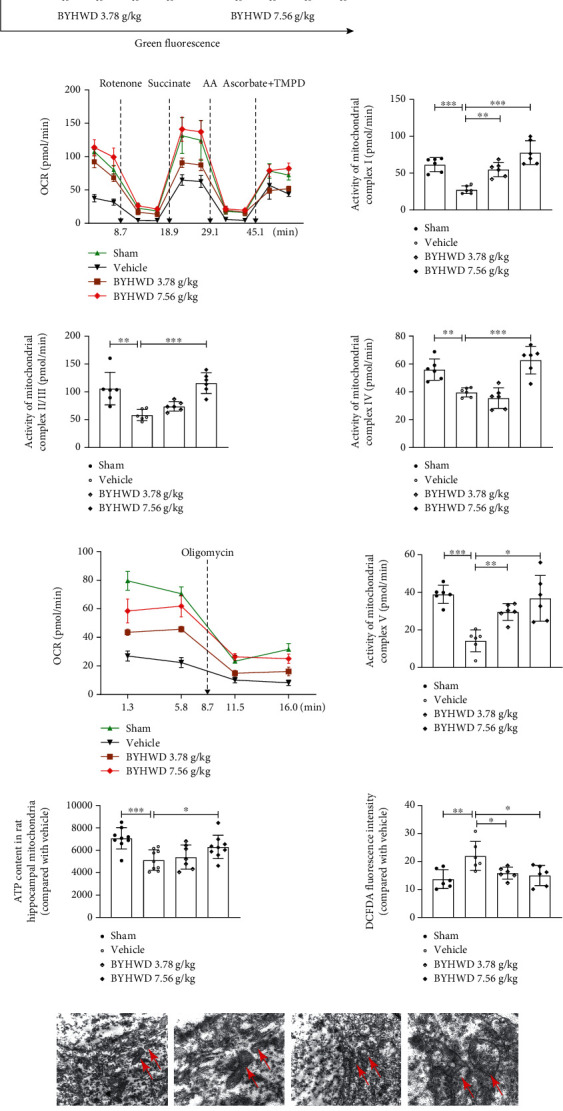
BYHW decoction alleviated CMI-induced mitochondrial injury in rat hippocampal neurons. (a) Representative flow cytometry data of mitochondrial membrane potential (Δ*ψm*) in cocultures with JC-1. JC-1 fluorescence is displayed as a red-to-green ratio, where the polymeric form is red and indicates a higher membrane potential, and the monomeric form is green and indicates a lower membrane potential (*n* = 3 rats). (b) Mitochondrial complex activity was measured by OCR in the freshly isolated mitochondria from the hippocampi of rats. Variation of OCR before and after injection of rotenone, succinate, antimycin A (AA), and ascorbate+TMPD revealed the activity of complex I (between 5.8 and 16 min), complex II/III (between 16 and 26.2 min), and complex IV (between 36.5 and 46.6 min) (*n* = 3 rats). (c) Isolated mitochondria were incubated in MAS solution with rotenone, succinic acid, and ADP. The activity of complex V was evaluated by variation of OCR before and after injection of oligomycin, a complex V inhibitor, between 5.8 and 16 min (*n* = 3 rats). ATP and ROS levels in the hippocampus were measured using the ATPlite kit (*n* = 3 rats) and H2DCFDA fluorescence probe (*n* = 3 rats). (d) Representative images from ultrastructural examination of ischemic neurons in the hippocampus. Mitochondria are marked with red arrows in the 30,000× images and are shown enlarged in the 60,000× images (*n* = 3 rats). The statistical comparisons were performed with one-way ANOVA analysis using GraphPad Prism. ^∗^*P* < 0.05, ^∗∗^*P* < 0.01, and ^∗∗∗^*P* < 0.001 versus the Vehicle group.

Mitochondrial complexes are known to undergo damage following an ischemic insult, leading to energy depletion, excessive ROS generation, and apoptosis. We examined the mitochondrial membrane potential (Δ*ψm*) and mitochondrial complex activity in CMI-injured rats to explore the effects of the BYHW decoction on mitochondrial function. Rats that were injured by CMIs showed lower Δ*ψm* compared with those in the Sham group. 7.56 g/kg BYHW decoction administration increased the Δ*ψm* by 1.51 times that of the Vehicle group (Figure 5(a)). Furthermore, we isolated the mitochondria from rat hippocampi and measured their OCR with a Seahorse XF96 analyzer. Using various mitochondrial complex substrates and inhibitors, we found that CMIs triggered a marked decline in complex activity. Specifically, complex I activity in the BYHW decoction treatment groups was 1.0–1.8 times higher than that of the Vehicle group (Figure 5(b)). Complexes II/III and IV also significantly improved their respiratory function following treatment with 7.56 g/kg BYHW decoction (Figure 5(b)). We also found that the activity of complex V was 1.1–1.6 times greater in the BYHW decoction groups compared with the Vehicle group (Figure 5(c)). ATP and ROS production reflects the function of mitochondrial complexes, and the resulting data is consistent with the mitochondrial complexes (Figure 5(c)).

In addition, changes in mitochondrial morphology that were observed in the Vehicle group were reversed in the groups treated with BYHW decoction; specifically, the mitochondrial cristae were greater in number and clearer in appearance (Figure 5(d)).

Collectively, these data revealed that the neuroprotection provided by BYHW decoction against CMI-induced apoptosis may involve its protective effects on mitochondrial complex function.

### 3.5. Regulated Effects of BYHW Decoction in the Hippocampus Assessed Using Proteomic Technologies and Parallel Reaction Monitoring (PRM)

**Figure 6:**
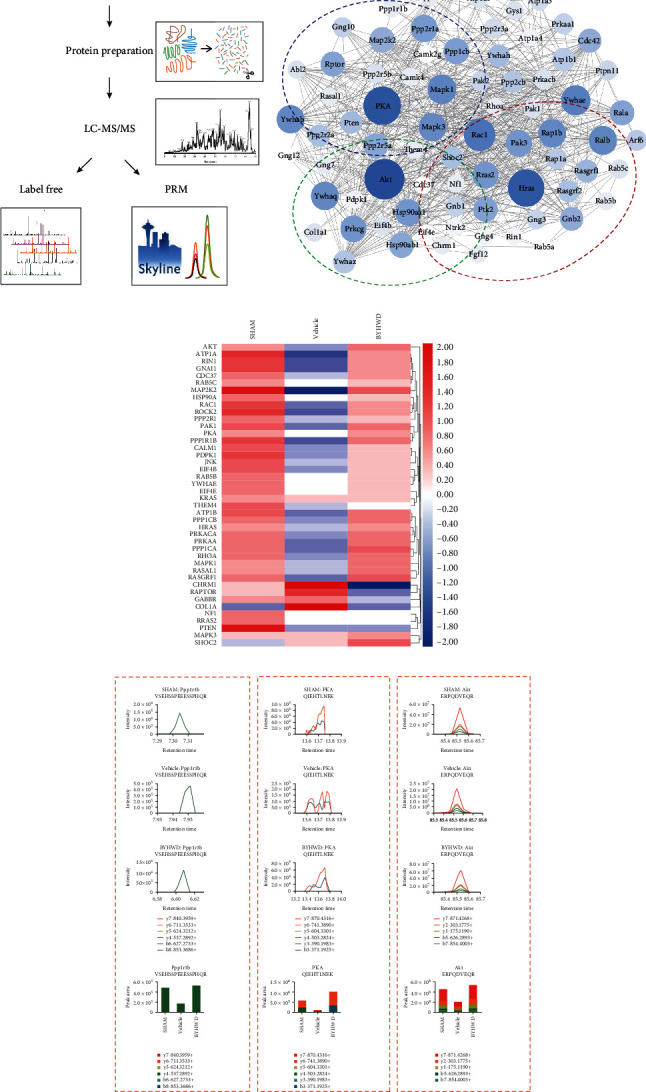
Effects of BYHW decoction on the regulation of signaling pathways assessed by proteomic analysis and parallel reaction monitoring (PRM). (a) Workflow: following 6 weeks of BYHW decoction treatment, quantitative proteomics was employed to profile the global proteomes of hippocampal cells in the different groups. Hippocampi were isolated from the rat brains, digested using trypsin, and subsequently subjected to UPLC-MS/MS. The resulting UPLC-MS/MS data were used to identify peptides (inferred to proteins) present in the samples. Finally, label-free and PRM analyses were conducted to verify the initial results and confirm the expression levels of the proteins of interest. (b) Protein interaction network of the CREB-related DAPs analyzed using the STRING database. Here, the colored circles represent groups of genes in different pathways associated with CREB. Diagram of the protein interaction shows the network of CREB-related proteins, including cAMP-PKA (green circle), Ras-MAPK (red circle), and PI3K-AKT (blue circle). The different sizes (from small to large) and shades (from light to dark) of the gene circles represent the degree of regulation by BYHW decoction (from weak to strong). (c) Heat map showing the expression levels of CREB-related proteins in different groups. Color depth represents expression differences between groups ranging from red (upregulated) to blue (downregulated). (d) Expression profiles of specific CREB-related proteins (specifically, Ppp1r1b, PKA, and AKT) were confirmed using PRM. Specific peptides of the proteins from different groups were determined, which can be used to predict the target gene. Columns of the proteins from different groups were generated by integrating all the peak areas of the precursor ions (*n* = 3 rats).

To elucidate the regulatory mechanisms by which the BYHW decoction reversed mitochondrial complex dysfunction, we engaged proteomic technologies to investigate changes in protein expression in the rat hippocampus among the Sham, Vehicle, and 7.56 g/kg BYHW decoction groups (Figure 6(a)). We used label-free quantitative proteomics technology to survey the global changes in hippocampal proteins. Among the 3500 proteins that we detected in the hippocampus, 823 were identified as different abundant proteins (DAPs), which were altered by CMIs at least 1.5-fold and recovered by BYHW decoction treatment. Bioinformatics and functional enrichment analyses of the DAPs revealed several biological processes. Among them, CREB-related proteins were found to be closely regulated by the BYHW decoction. The heat map shows these CREB-related signaling pathways, including cAMP-PKA, Ras-MAPK, and PI3K-AKT pathways (Figure 6(b)).  Figure 6(c) also depicts that the protein clusters of PKA, AKT, and MAPK signaling pathway-related proteins were downregulated in the Vehicle group and that BYHW decoction treatment reversed the decline.

Additionally, we conducted PRM, a targeted proteomic study, to validate the expression of CREB-associated proteins. Consistent with our proteomics results, PRM revealed that the expression of PKA, AKT, and Ppp1r, as well as cAMP-PKA and PI3K-AKT pathway-related proteins was altered in our CMI model, and expression levels recovered after BYHW decoction treatment (Figure 6(d)). These data strengthened our hypothesis that the BYHW decoction acts through the PKA/CREB pathway to modulate its effects on mitochondrial function.

### 3.6. Effects of BYHW Decoction on the PKA/CREB Signaling Pathway

**Figure 7:**
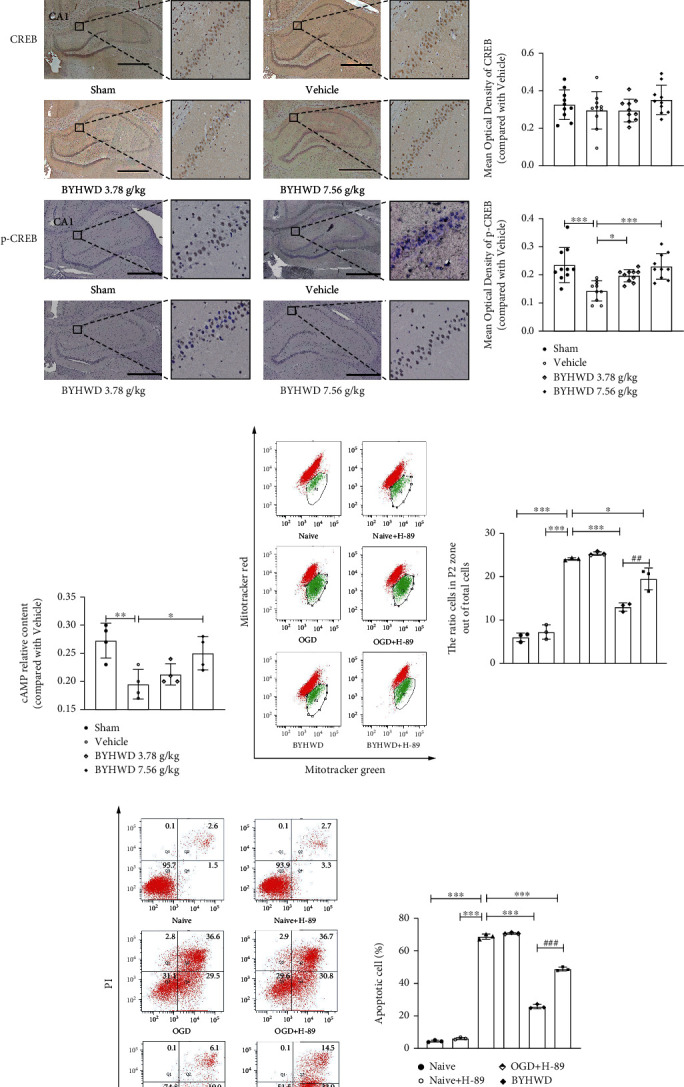
Effects of BYHW decoction on activity and expression for CREB-related proteins as determined by immunohistochemistry (a, b). Immunohistochemical visualization and quantification of PKA and CREB in the rat hippocampus (scale bar, 1 mm). The average optical density of CREB and PKA in the hippocampus (*n* = 3 rats). (c) CAMP content from the hippocampus was determined by ELISA kits. ^∗^*P* < 0.05 and ^∗∗^*P* < 0.01 versus the Vehicle group (*n* = 4 rats). (d–g) Effects of BYHW decoction on mitochondrial damage (MitoTracker red and green), (e) neuronal apoptosis (Annexin V-FITC/PI), and ROS and ATP generation with or without H-89. *n* = 3. ^∗^*P* < 0.05, ^∗∗^*P* < 0.01, and ^∗∗∗^*P* < 0.001 versus the Vehicle group. ^##^*P* < 0.01 and ^###^*P* < 0.001 compared with the BYHWD+H-89 group.

To experimentally test the influence of the BYHW decoction on the PKA/CREB pathway, we determined the expression and activity of PKA and CREB in our various treatment groups. Immunohistochemical detection revealed an upregulation of PKA expression and activity (p-PKA) following BYHW decoction treatments compared with expression levels in the CMI-injured group. PKA is mainly expressed in the cytoplasm and synapse, as well as in the mitochondria [17]. We found that CMIs induced a decline in PKA expression and activity in the neuronal cytoplasm and synapse, which was reversed by BYHW decoction treatment (Figure 7(a)). Unlike PKA, the cellular distribution of CREB includes both the cytoplasm and the nucleus, as well as the mitochondria [18]. We found that there was no significant difference in CREB expression between the Vehicle and other groups (Figure 7(b)). However, rats that were injured by CMIs showed lower activity of CREB (p-CREB) compared with those in the Sham group. BYHW decoction administration partially reversed the decline, indicating that the activity of CREB is regulated by PKA and BYHW decoction may protect the mitochondria by activating the PKA/CREB pathway. Moreover, we found that the level of cAMP, a molecule upstream of PKA, also increased following BYHW decoction treatment (Figure 7(c)).

We have shown above that BYHW decoction alleviated mitochondrial injury caused by CMIs, and that CMI-induced decline in PKA and CREB activity was reversed by BYHW decoction treatment. Thus, we hypothesized that the beneficial effects of BYHW decoction were induced by derepression of the PKA/CREB signaling pathway. In support of this hypothesis, the PKA inhibitor, H-89, partially suppressed the effects of BYHW decoction on neuronal apoptosis and mitochondrial damage, as well as ATP and ROS content in vitro, suggesting that PKA/CREB signaling directly modulated mitochondrial activity following BYHW decoction treatment (Figures 7(d)–7(g)).

Taken together, the data from the proteomic analyses, immunohistochemistry, and flow cytometry demonstrated that the mechanisms underlying the neuroprotective effects of BYHW decoction treatment after CMIs include the activation of the PKA/CREB signaling pathways in the mitochondria of the rat hippocampus.

## 4. Discussion

CMIs refer to a group of small ischemic lesions associated with cognitive impairments and dementia [47]. Our study aimed at exploring the feasibility of this traditional Chinese remedy as a therapeutic strategy against refractory diseases such as CMIs. BYHW decoction, the most frequently used traditional Chinese treatment for stroke in China, has been reported to improve the cognition impairments following stroke [23]. BYHW decoction is widely used to treat stroke and poststroke conditions in China, and numerous animal and clinical trials have proved its effectiveness. One meta-analysis that assessed 56 studies with 1270 animals indicated a substantial neuroprotective action from BYHW decoction in models of focal cerebral ischemia [48]. The possible mechanism involved anti-inflammation [22], antioxidation [49], regulation of VEGF or angiopoietin-1 expression [50, 51], and improvement of synaptic plasticity [52]. Clinical studies have also proved the effectiveness of BYHW decoction on stroke [53–55]. Furthermore, BYHW decoction also showed beneficial effects on poststroke conditions [56] such as poststroke epilepsy [28], poststroke fatigue [57], and poststroke dementia [58]. The study provides novel evidence that BYHW decoction treatment may help patients with CMIs to gain benefits on recognitive and motor ability. Furthermore, we identified, herein, that the activation of PKA/CREB protected mitochondrial complexes, generated more ATP and less ROS, and finally reduced neuronal apoptosis as the key mechanism responsible for the CMI-protective properties of BYHW decoction in rats. Moreover, we used an in vitro model of ischemic injury via oxygen and glucose deprivation (OGD) of cultured primary neurons and confirmed that BYHW decoction significantly decreased the levels of ROS in mitochondria (Supplementary Figure 2).

Mitochondrial damage is a key event in the development of cerebral ischemic injury. The potential reason might involve conformational change of mitochondrial complex I. In fact, it is reported that there is a transition of the active mitochondrial complex I to the deactive form during ischemia in the brain, and the deactive complex I cannot generate enough ATP, even if it catalyzes more NADH to NAD+ [59, 60]. Moreover, there was even a positron emission tomography (PET) probe created to monitor the activity of mitochondrial complexes, in order to determine the pathological progression of ischemic stroke and evaluate the effects of potential therapeutic agents [61, 62]. In this study, we showed that BYHW decoction treatments reversed mitochondrial complex dysfunction in the rat hippocampus and increased ATP production, which contributed to increased neuronal survival and improved cognitive function.

Using quantitative proteomics, we noticed that the proteins associated with the PKA/CREB pathway were influenced by the BYHW decoction. This was consistent with its beneficial effects, as numerous animal studies have shown an essential role of CREB in memory and cognitive function [13, 14] and that PKA is an upstream kinase that modulates CREB activity. We showed that treatment with the BYHW decoction upregulated the expression of PKA and CREB and other proteins in the PKA/CREB pathway. The results in vitro demonstrated that the protective effects of BYHW decoction on apoptosis, mitochondria damage, and ATP and ROS content were partially blocked by PKA inhibitor H-89, indicating that BYHW decoction protected the mitochondria by activating the PKA/CREB pathway (Figures 7(d)–7(g)). However, it has been well reported that the regulatory kinases of CREB include not only PKA, but also MAPK, AKT, and CaMKII [63]. Consistently, our proteomics analyses revealed that AKT and MAPK pathways were also regulated by treatment with the BYHW decoction (Figure 5(a)). Furthermore, we used PRM to verify the modulation of CREB-related protein AKT by the BYHW decoction (Figure 5(b)). Since the AKT/CREB pathway was also highlighted by our proteomics and PRM analyses, it represents another potential mechanism underlying the effects of the BYHW decoction. This speculation requires further verification. Collectively, our results indicate that PKA/CREB is one of the pathways altered by BYHW decoction but not the only one.

There are four genes that showed a reversed expression level compared to all the rest, namely, CHRM1, RAPTOR, GABBR, and COL1A, which increased in the Vehicle group compared to the Sham group and decreased following BYHW decoction treatment for 6 weeks, as shown in Figures 5(b) and 5(c). These genes are involved in protein synthesis and cognitive function [64–67]. It is possible that the expression of these genes increased sharply in the CMI-injured group due to the regulation of the body under ischemic pressure and they lasted a long time (at least 6 weeks). However, following 6 weeks of treatment, these gene expressions in the BYHW decoction group decreased maybe because BYHW decoction treatment changed the condition of ischemic pressure and cerebral injury. In future studies, we will verify these interesting results and examine the specific effect of BYHW decoction on these atypical genes.

Our data revealed that cAMP levels also increased after BYHW decoction treatment compared to the Vehicle-treated rats (Figure 6(d)). This result increases the complexity of the mechanisms underlying the BYHW decoction's effects. Since PKA is a cAMP-dependent kinase whose activity relies on cellular levels of cAMP, the above finding suggests that the PKA/CREB-mediated effects of BYHW decoction in CMIs may partially stem from the effect of the treatment on cAMP. Similarly, it is possible that the BYHW decoction does not directly act on PKA or AKT but instead interacts with upstream proteins such as adenylyl cyclase (AC), the G_s_-protein or inhibiting PDE (phosphodiesterase) to regulate the levels of cAMP, which in turn influence PKA and CREB activity [68, 69]. However, more focused experiments are required to confirm the precise point of action of the BYHW decoction.

## Figures and Tables

**Table 1:** Linear range, *R*^2^, and limits of quantification of calibration curve used to determine the main identified compounds.

Compounds	Linear range (ng)	Calibration curve	*R* ^2^
Amygdalin	0.2-1000	*Y* = 1.39^∗^10^4^*X* − 359	0.9978
Hydroxysafflor yellow A	0.2-1000	*Y* = 4.76^∗^10^3^*X* − 2.24^∗^10^3^	0.9908
Paeoniflorin	0.2-1000	*Y* = 1.33^∗^10^4^*X* − 1.77^∗^10^3^	0.9962
Ferulic acid	0.2-1000	*Y* = 2.27^∗^10^3^*X* − 298	0.9978
Senkyunolide I	0.2-1000	*Y* = 2.04^∗^10^4^*X* + 9.28^∗^10^3^	0.9952
Senkyunolide H	0.2-1000	*Y* = 2.67^∗^10^4^*X* − 4.22^∗^10^3^	0.9962
Benzoylpaeoniflorin	0.2-1000	*Y* = 3.30^∗^10^4^*X* − 3.42^∗^10^3^	0.9970
Formononetin	0.2-200	*Y* = 4.69^∗^10^5^*X* − 9.16^∗^10^3^	0.9990
Astragaloside IV	75-1000	*Y* = 25.4*X* − 390	0.9939

**Table 2:** Contents of the main identified compounds in BYHW decoction.

Herbs	Compounds	Contents (*μ*g/g)
*Astragalus membranaceus* Fisch. (Huang Qi)	Formononetin	217.05
	Astragaloside IV	4234.37
*Ligusticum sinense* Oliv. (Chuan Xiong)	Senkyunolide H	26.18
	Senkyunolide I	155.18
*Angelica sinensis* (Oliv.) Diels (Dang Gui)	Ferulic acid	19.05
*Carthamus tinctorius* L. (Hong Hua)	Hydroxysafflor yellow A	19.40
*Paeonia lactiflora* Pall. (Chi Shao)	Benzoylpaeoniflorin	905.14
	Paeoniflorin	48.50
*Prunus persica* (L.) Stokes (Tao Ren)	Amygdalin	1580.50

## Data Availability

The datasets generated during and/or analyzed during the current study are available from the author (xuelibang2008@163.com) on reasonable request.

## Supplementary Materials

Supplementary Materials Graphical abstract. In this study, we applied microsphere-induced cerebral microinfarct (CMI) model in rats to investigate the behavioral and molecular consequences of CMIs. We used the Morris water maze, quantitative proteomics, and other molecular assays and found that activation of the PKA/CREB pathway by BYHW decoction treatment may reverse mitochondrial dysfunction, regulate the generation of ROS and ATP, inhibit apoptosis of hippocampal neurons, and ameliorate CMI-induced cognitive impairments in rats. Collectively, these findings confirmed the therapeutic potential of the BYHW decoction in treating cognitive impairments induced by CMIs and demonstrated a viable mechanism for its action. Supplementary Figure 1: original image of MS1 and MS2 in the chromatograms of the main identified components of BYHW decoction, including Amygdalin, Hydroxysaffor yellow A, Paeoniflorin, Ferulic acid, Senkyunolide I, Senkyunolide H, Benzoylpaeoniflorin, Formononetin, and Astragaloside IV. Supplementary Table 1: a gradient eluting program in UPLC (A) and parameters in MS (B). Supplementary Figure 2: ROS production was measured in primary hippocampal neurons with MitoSOX Red staining, with Hoechst 33342 to label all cell nuclei. Representative images are shown (magnification, 20×), image in each inset. The quantified levels of ROS are expressed as the means ± SD. *n* = 5. ^∗^*P* < 0.05 and ^∗∗∗^*P* < 0.001 compared with the OGD group.
