# Supplementary material for: BYHW Decoction Improves Cognitive Impairments in Rats with Cerebral Microinfarcts via Activation of the PKA/CREB Pathway
Source: Oxid Med Cell Longev. 2022 Dec 30;2022:4455654. doi: 10.1155/2022/4455654 (PMC9822752; doi:10.1155/2022/4455654)
Supplement: Supplementary Materials — Graphical abstract. In this study, we applied microsphere-induced cerebral microinfarct (CMI) model in rats to investigate the behavioral and molecular consequences of CMIs. We used the Morris water maze, quantitative proteomics, and other molecular assays and found that activation of the PKA/CREB pathway by BYHW decoction treatment may reverse mitochondrial dysfunction, regulate the generation of ROS and ATP, inhibit apoptosis of hippocampal neurons, and ameliorate CMI-induced cognitive impairments in rats. Collectively, these findings confirmed the therapeutic potential of the BYHW decoction in treating cognitive impairments induced by CMIs and demonstrated a viable mechanism for its action. Supplementary Figure 1: original image of MS1 and MS2 in the chromatograms of the main identified components of BYHW decoction, including Amygdalin, Hydroxysaffor yellow A, Paeoniflorin, Ferulic acid, Senkyunolide I, Senkyunolide H, Benzoylpaeoniflorin, Formononetin, and Astragaloside IV. Supplementary Table 1: a gradient eluting program in UPLC (A) and parameters in MS (B). Supplementary Figure 2: ROS production was measured in primary hippocampal neurons with MitoSOX Red staining, with Hoechst 33342 to label all cell nuclei. Representative images are shown (magnification, 20×), image in each inset. The quantified levels of ROS are expressed as the means ± SD. n = 5. ∗P < 0.05 and ∗∗∗P < 0.001 compared with the OGD group. [file 4455654.f1.zip › Supplementary Figure 1 editable.pdf]

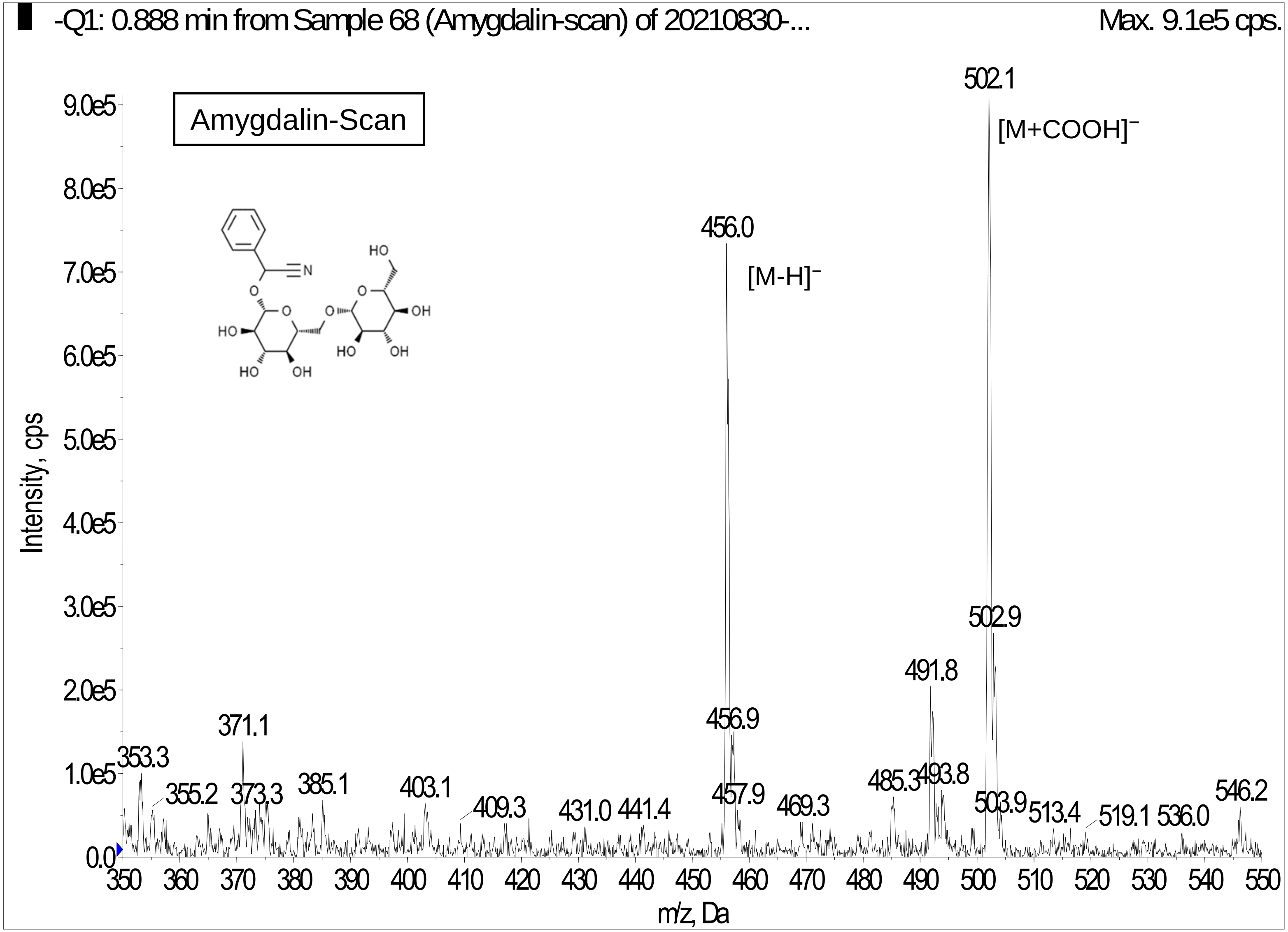

-Q1: 0.888 min from Sample 68 (Amygdalin-scan) of 20210830-...
Max. 9.1e5 cps.
Amygdalin-Scan
502.1
[M+COOH]⁻
456.0
[M-H]⁻
502.9
491.8
456.9
371.1
353.3
355.2
373.3
385.1
403.1
409.3
431.0
441.4
457.9
469.3
485.3
493.8
503.9
513.4
519.1
536.0
546.2
Intensity, cps
m/z, Da

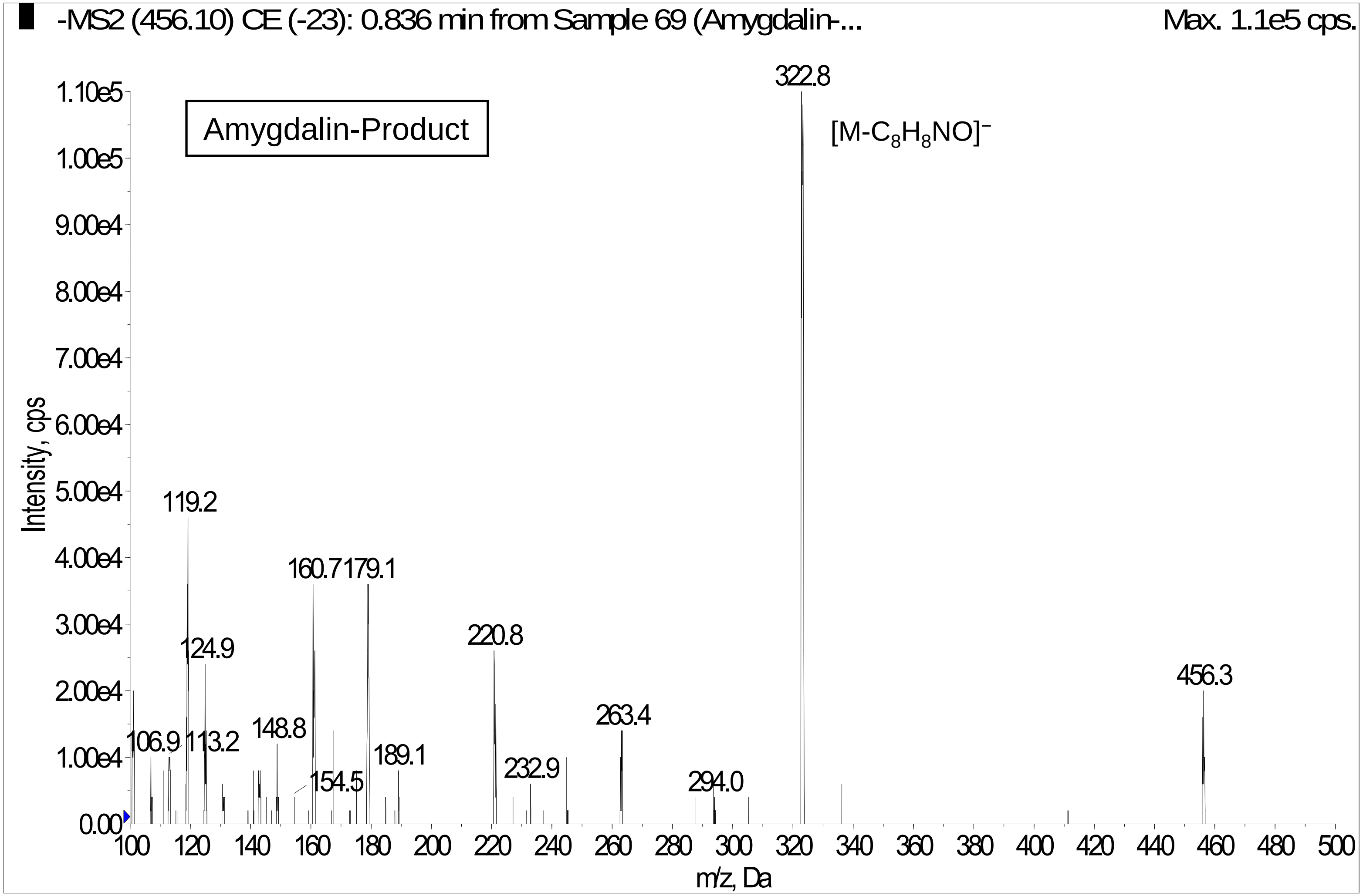

-MS2 (456.10) CE (-23): 0.836 min from Sample 69 (Amygdalin-...
Max. 1.1e5 cps.
Amygdalin-Product
[M-C8H8NO]⁻
322.8
119.2
160.7
179.1
124.9
220.8
456.3
263.4
148.8
106.9
113.2
189.1
232.9
154.5
294.0
Intensity, cps
m/z, Da
1.10e5
1.00e5
9.00e4
8.00e4
7.00e4
6.00e4
5.00e4
4.00e4
3.00e4
2.00e4
1.00e4
0.00
100 120 140 160 180 200 220 240 260 280 300 320 340 360 380 400 420 440 460 480 500

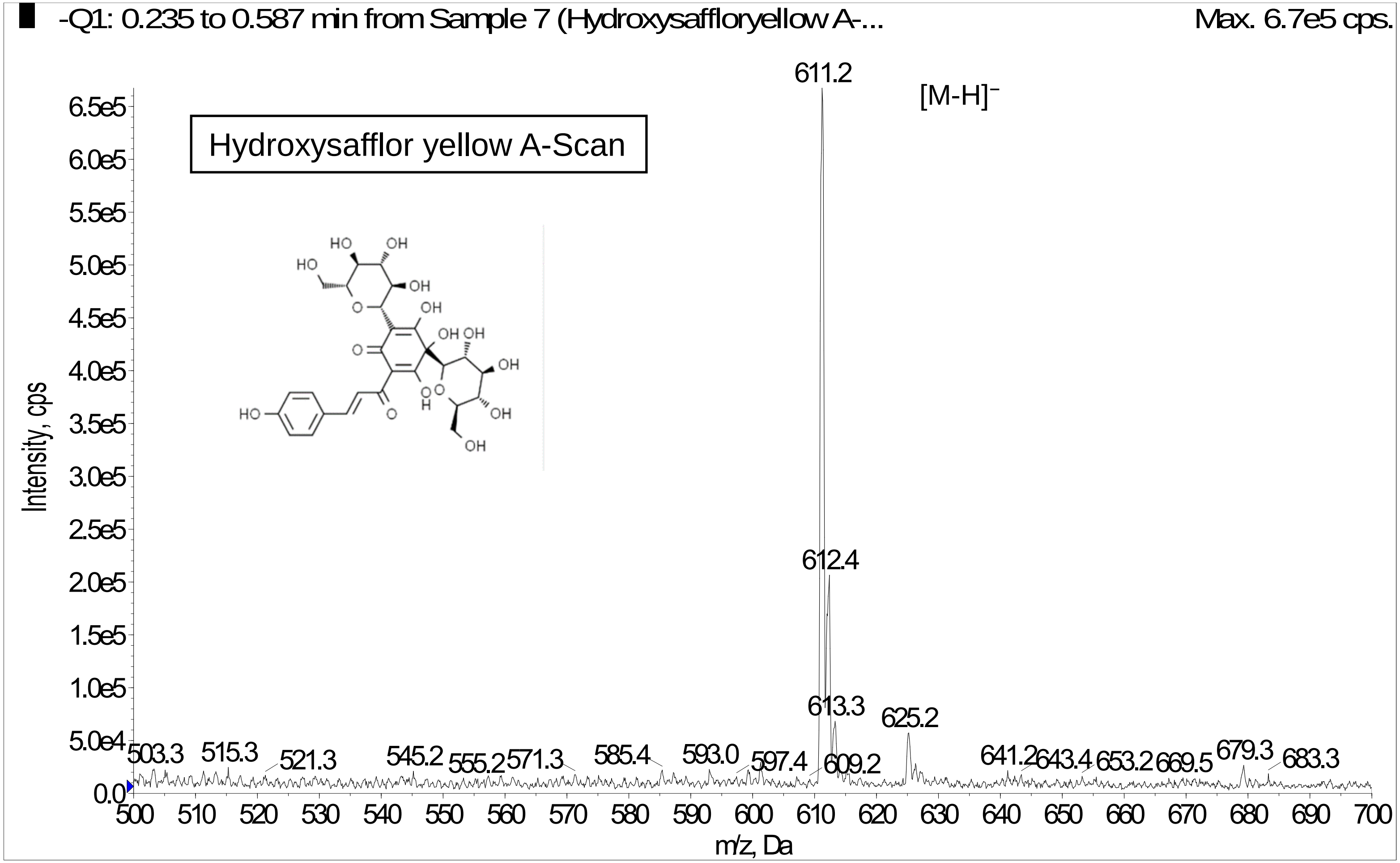
-Q1: 0.235 to 0.587 min from Sample 7 (Hydroxysafflor yellow A-...
Max. 6.7e5 cps.
Hydroxysafflor yellow A-Scan
611.2
[M-H]⁻
612.4
613.3
625.2
503.3
515.3
521.3
545.2
555.2
571.3
585.4
593.0
597.4
609.2
641.2
643.4
653.2
669.5
679.3
683.3
Intensity, cps
m/z, Da

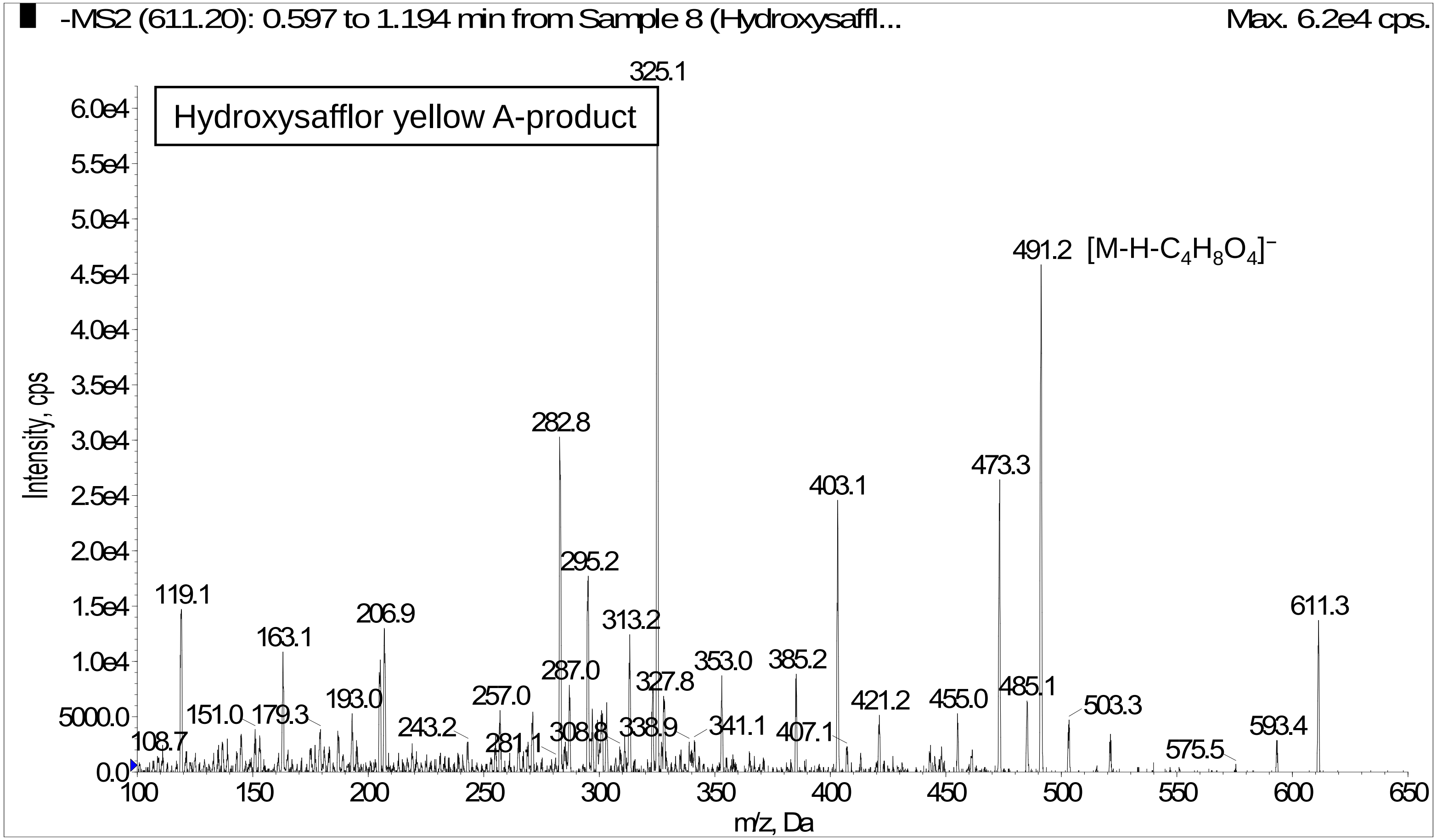

-MS2 (611.20): 0.597 to 1.194 min from Sample 8 (Hydroxysaffl...
Max. 6.2e4 cps.
Hydroxysafflor yellow A-product
491.2 [M-H-$C_4H_8O_4$]$^-$
325.1
282.8
473.3
403.1
295.2
119.1
206.9
163.1
313.2
611.3
287.0
353.0
385.2
327.8
257.0
193.0
421.2
455.0
485.1
503.3
151.0
179.3
243.2
108.7
281.1
308.8
338.9
341.1
407.1
575.5
593.4
Intensity, cps
m/z, Da

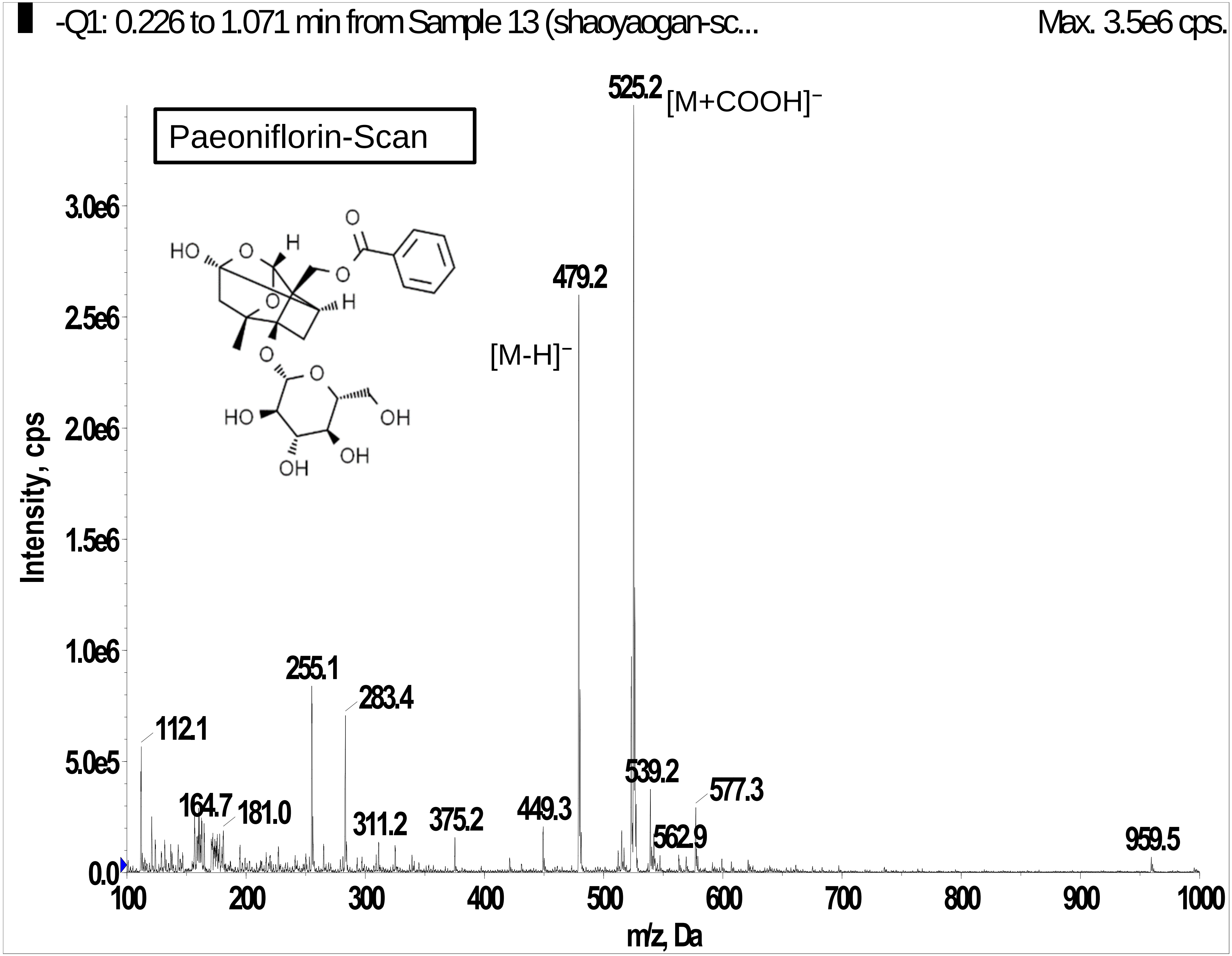

-Q1: 0.226 to 1.071 min from Sample 13 (shaoyaogan-sc...
Max. 3.5e6 cps.
Paeoniflorin-Scan
525.2
[M+COOH]⁻
479.2
[M-H]⁻
255.1
283.4
112.1
164.7
181.0
311.2
375.2
449.3
539.2
577.3
562.9
959.5
Intensity, cps
m/z, Da

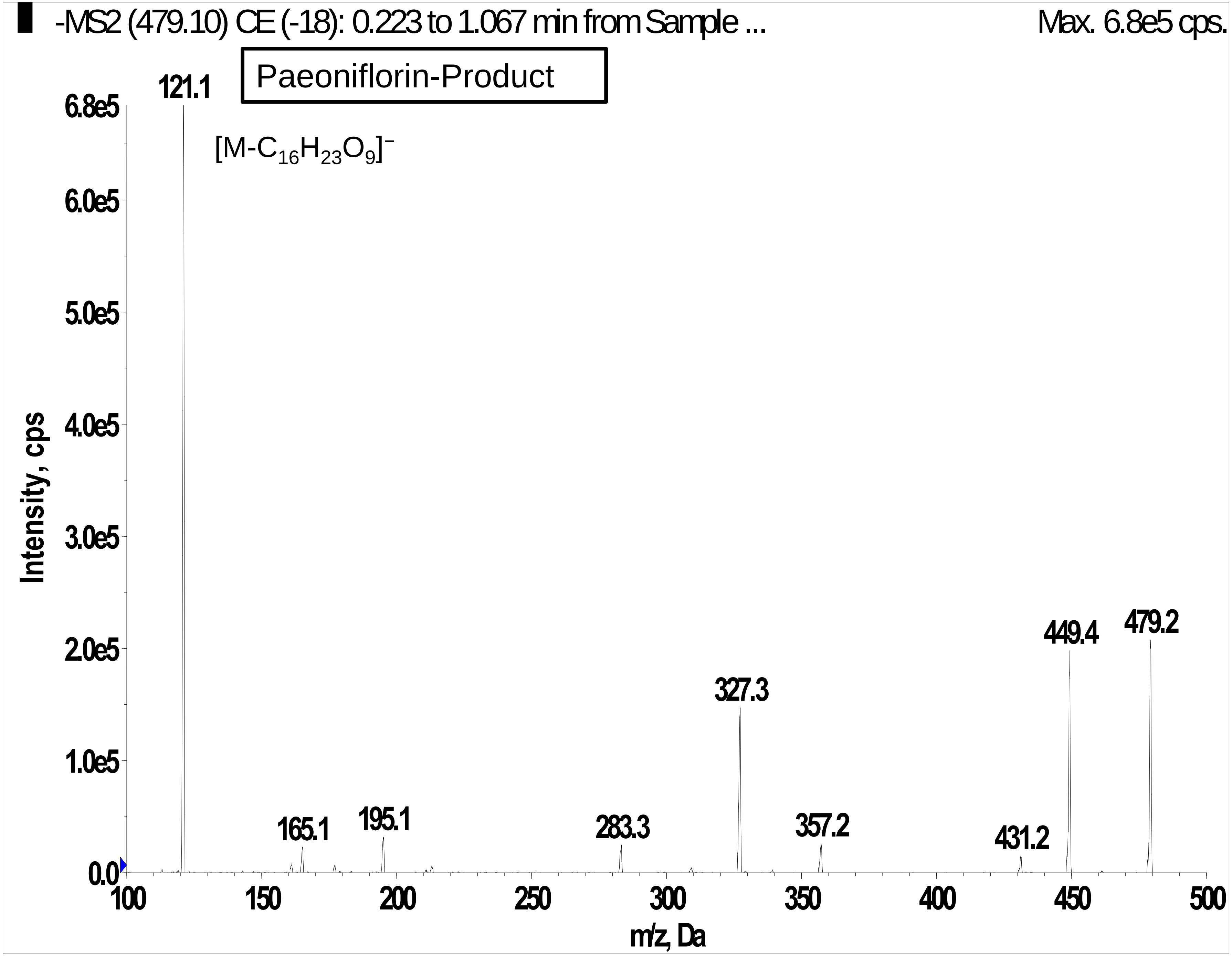

-MS2 (479.10) CE (-18): 0.223 to 1.067 min from Sample ...
Max. 6.8e5 cps.
Paeoniflorin-Product
[M-C16H23O9]⁻
121.1
165.1
195.1
283.3
327.3
357.2
431.2
449.4
479.2
Intensity, cps
6.8e5
6.0e5
5.0e5
4.0e5
3.0e5
2.0e5
1.0e5
0.0
100
150
200
250
300
350
400
450
500
m/z, Da

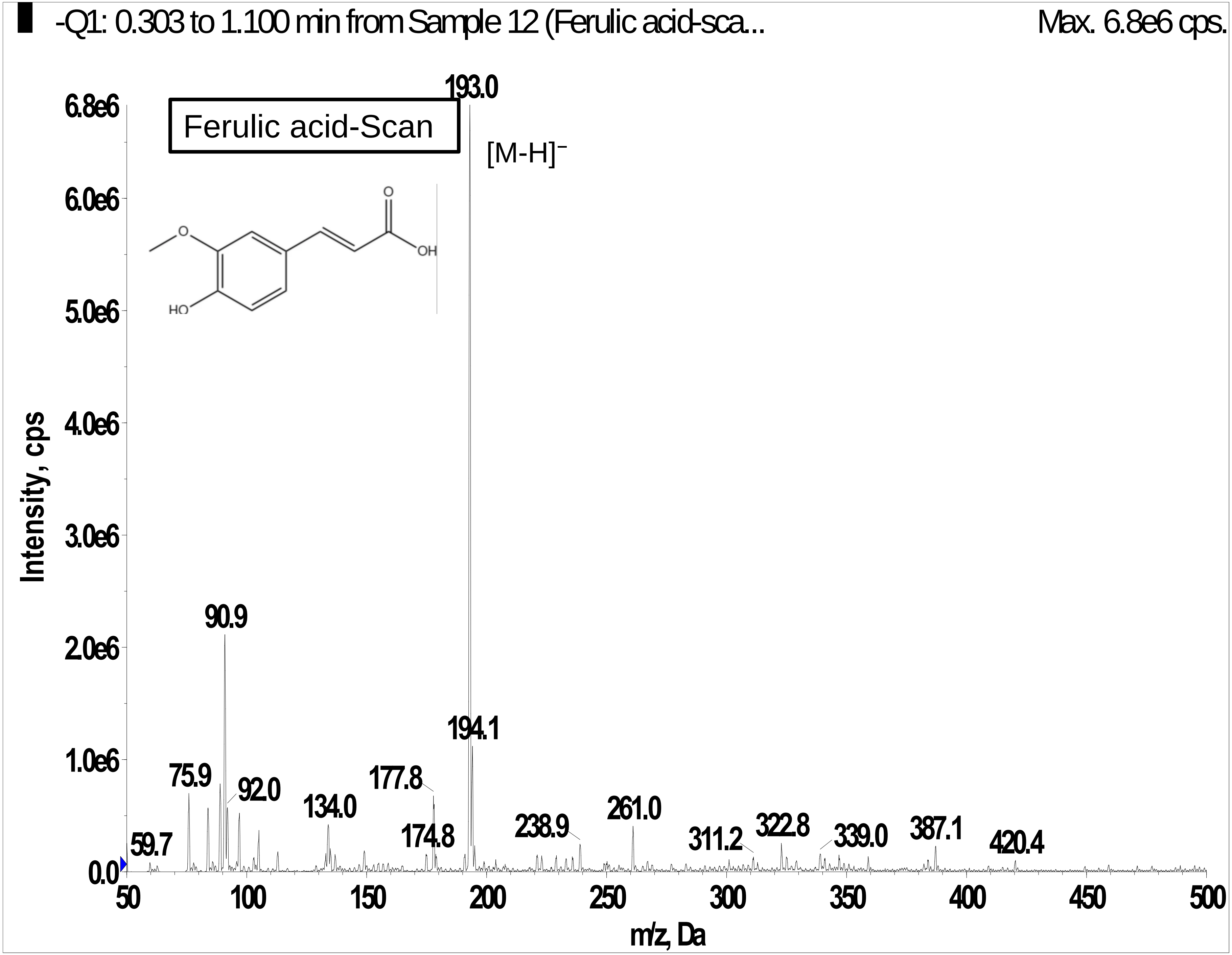
-Q1: 0.303 to 1.100 min from Sample 12 (Ferulic acid-sca...
Max. 6.8e6 cps.
Ferulic acid-Scan
[M-H]⁻
193.0
194.1
90.9
92.0
75.9
59.7
134.0
177.8
174.8
238.9
261.0
311.2
322.8
339.0
387.1
420.4
O
OH
HO
6.8e6
6.0e6
5.0e6
4.0e6
3.0e6
2.0e6
1.0e6
0.0
Intensity, cps
50
100
150
200
250
300
350
400
450
500
m/z, Da

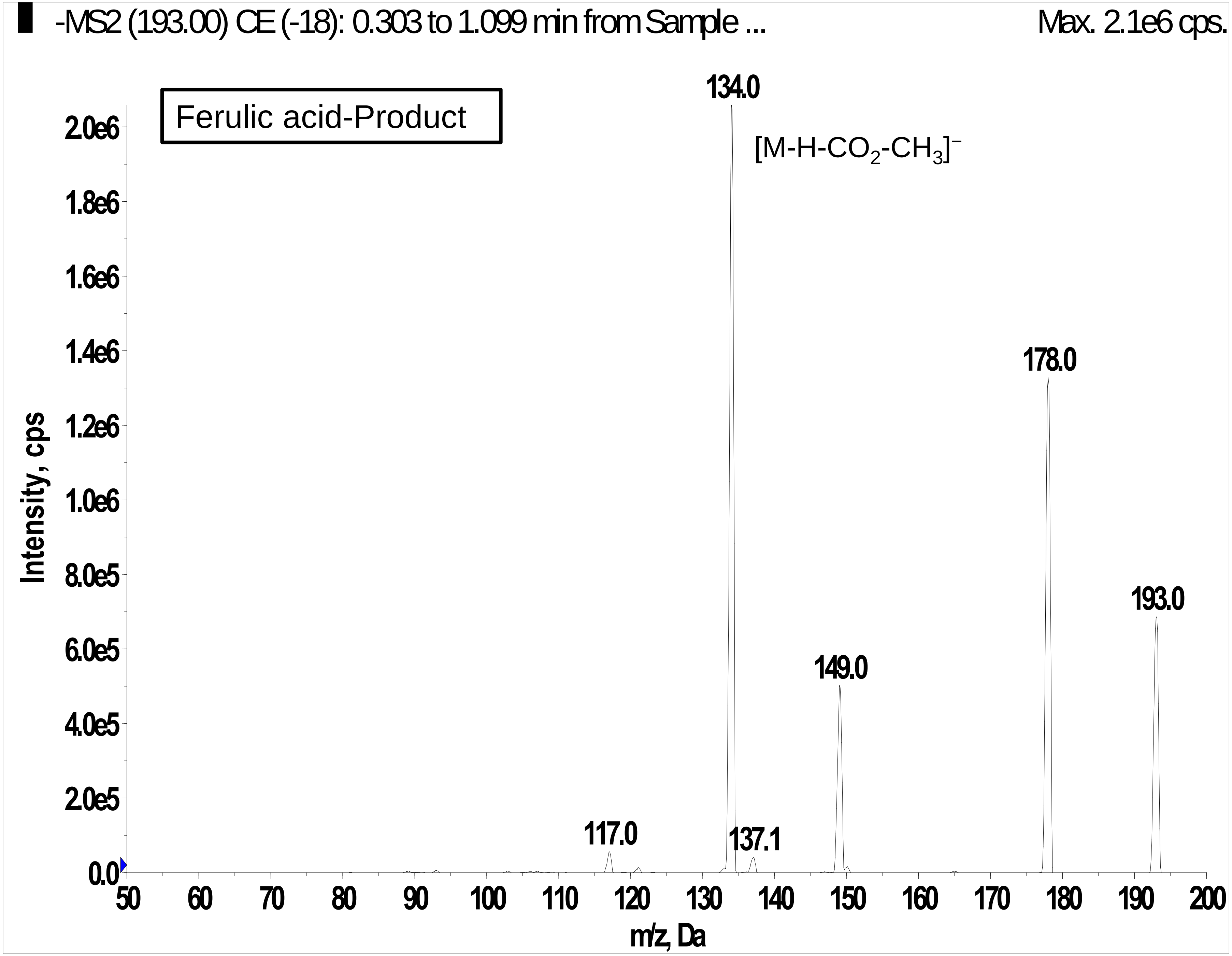
-MS2 (193.00) CE (-18): 0.303 to 1.099 min from Sample ...
Max. 2.1e6 cps.
Ferulic acid-Product
[M-H-CO2-CH3]⁻
134.0
178.0
193.0
149.0
117.0
137.1
Intensity, cps
2.0e6
1.8e6
1.6e6
1.4e6
1.2e6
1.0e6
8.0e5
6.0e5
4.0e5
2.0e5
0.0
50
60
70
80
90
100
110
120
130
140
150
160
170
180
190
200
m/z, Da

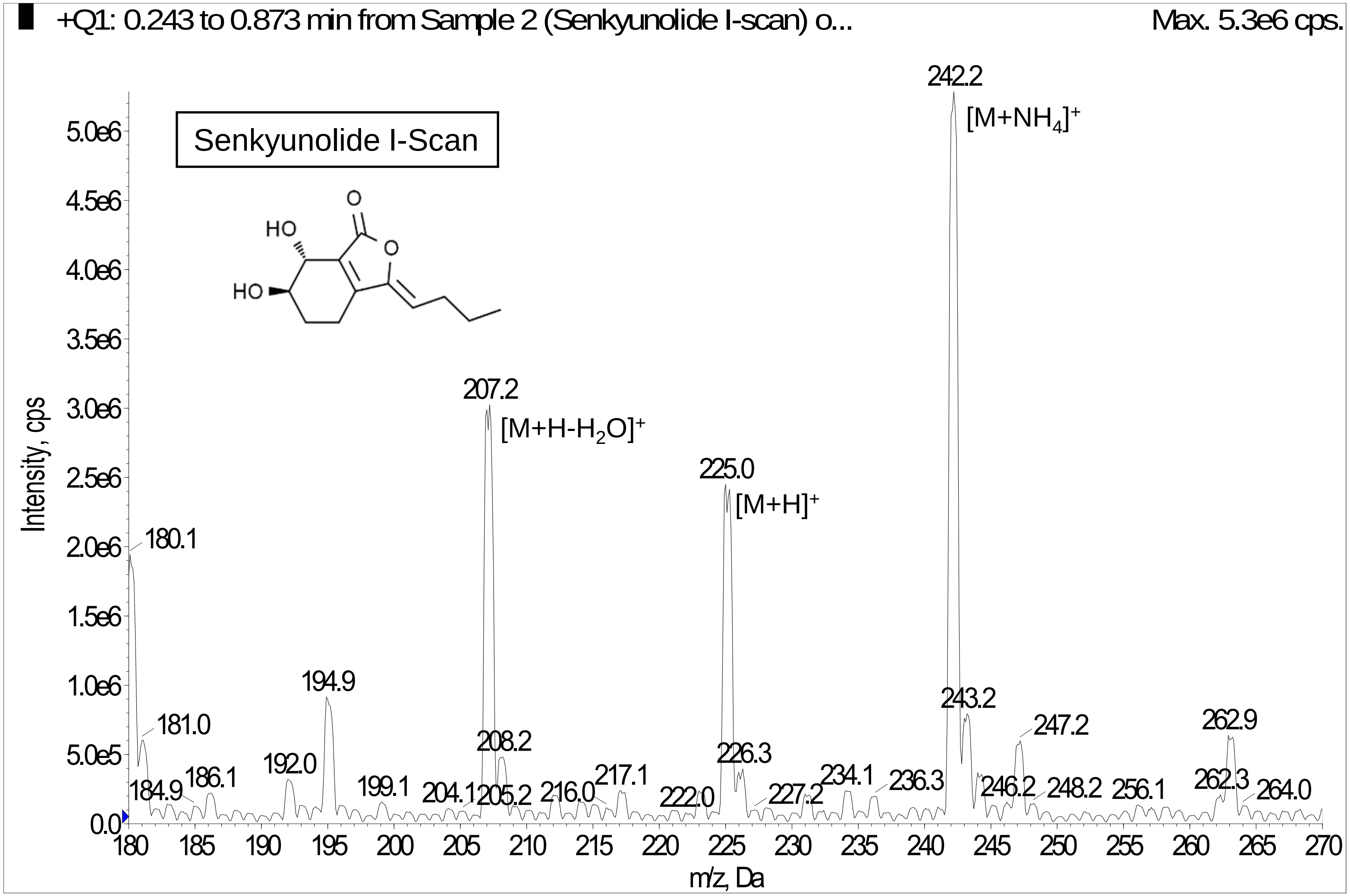

+Q1: 0.243 to 0.873 min from Sample 2 (Senkyunolide I-scan) o...
Max. 5.3e6 cps.
Senkyunolide I-Scan
HO
HO
O
O
242.2
[M+NH4]+
207.2
[M+H-H2O]+
225.0
[M+H]+
180.1
181.0
184.9
186.1
192.0
194.9
199.1
204.1
205.2
208.2
216.0
217.1
222.0
226.3
227.2
234.1
236.3
243.2
246.2
247.2
248.2
256.1
262.3
262.9
264.0
Intensity, cps
m/z, Da

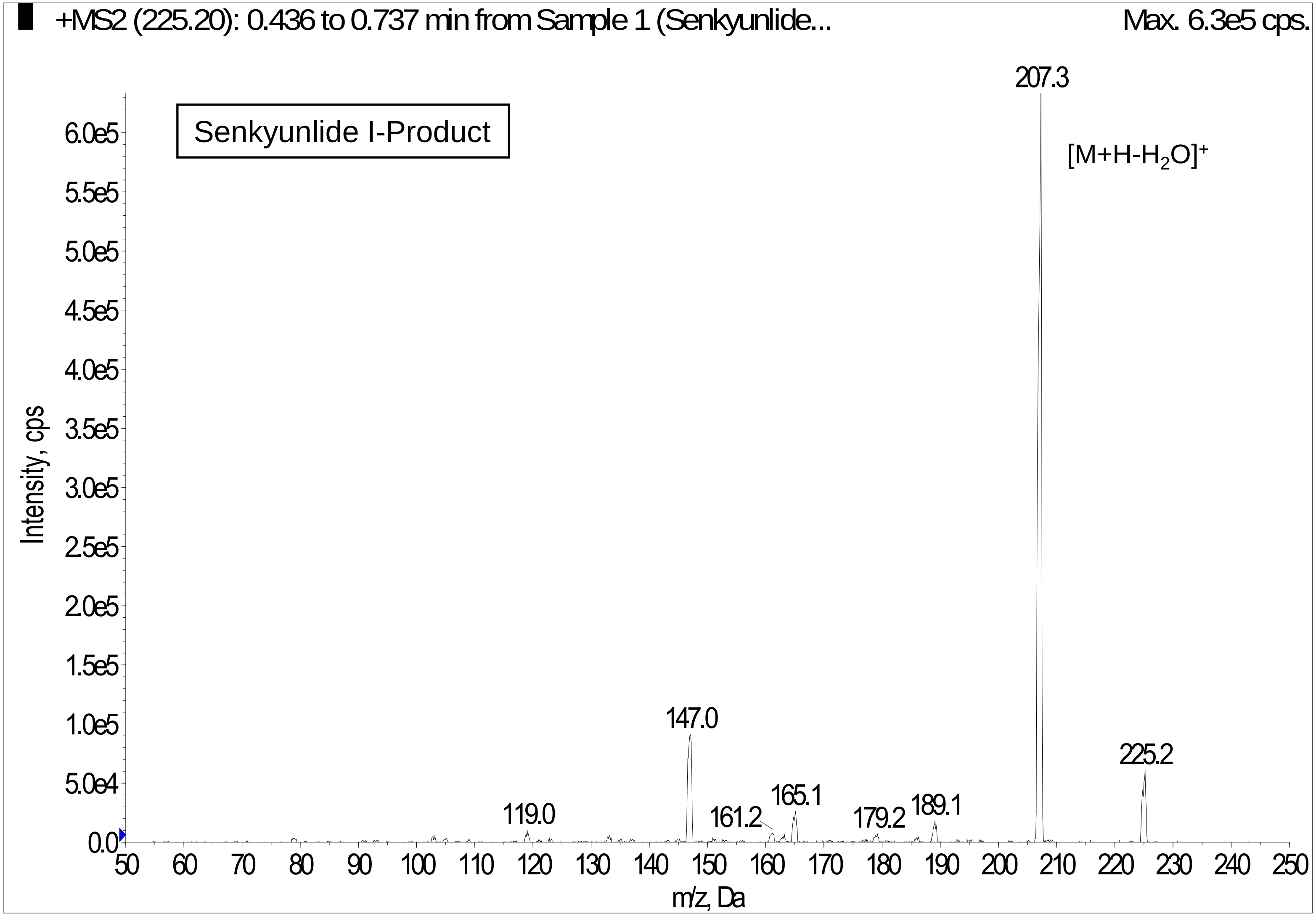
+MS2 (225.20): 0.436 to 0.737 min from Sample 1 (Senkyunlide...
Max. 6.3e5 cps.
Senkyunlide I-Product
207.3
[M+H-H2O]+
147.0
225.2
119.0
161.2
165.1
179.2
189.1
Intensity, cps
m/z, Da
6.0e5
5.5e5
5.0e5
4.5e5
4.0e5
3.5e5
3.0e5
2.5e5
2.0e5
1.5e5
1.0e5
5.0e4
0.0
50
60
70
80
90
100
110
120
130
140
150
160
170
180
190
200
210
220
230
240
250

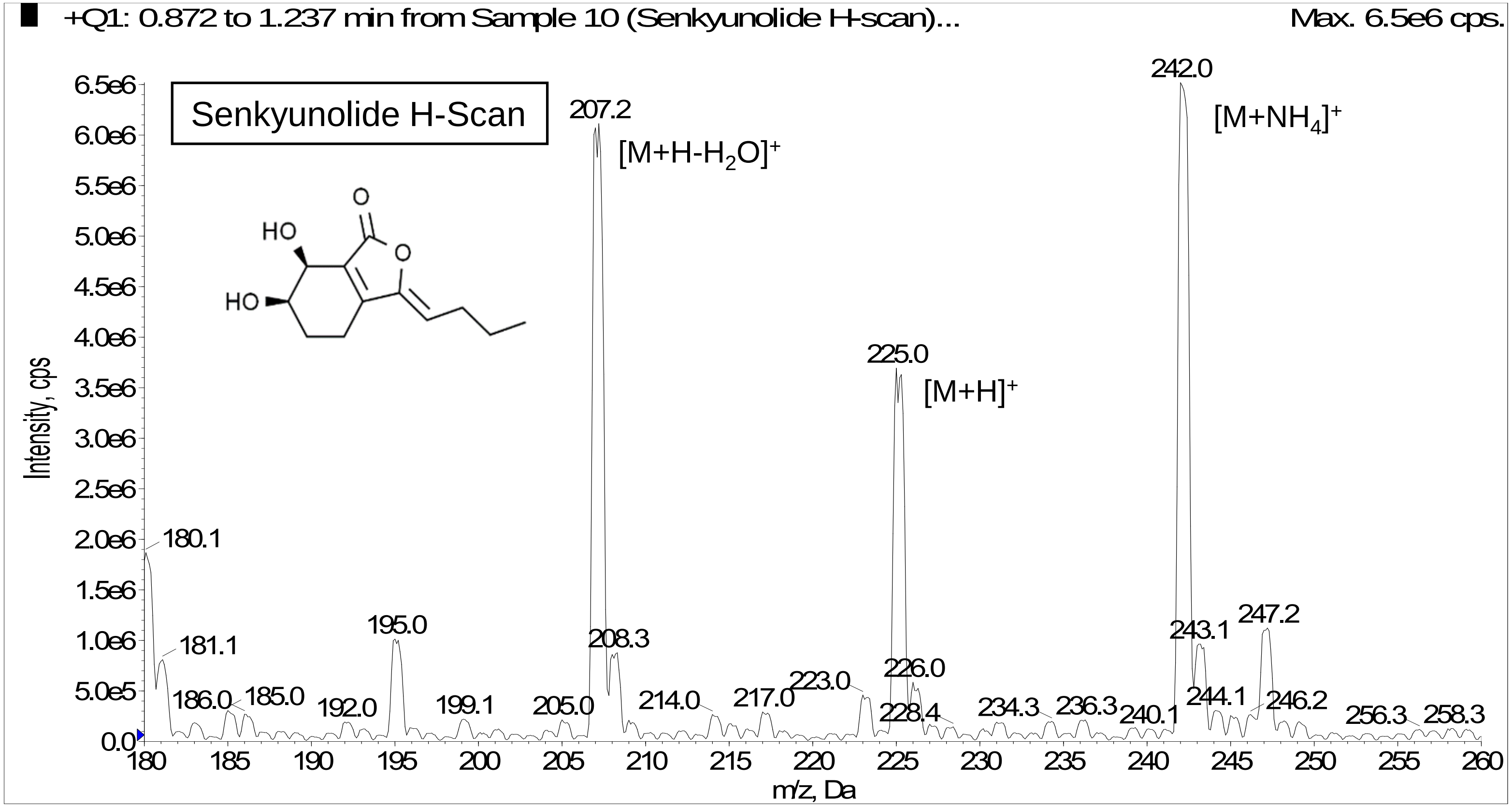
+Q1: 0.872 to 1.237 min from Sample 10 (Senkyunolide H-scan)...
Max. 6.5e6 cps.
Senkyunolide H-Scan
HO
HO
O
O
242.0
[M+NH4]+
207.2
[M+H-H2O]+
225.0
[M+H]+
180.1
181.1
186.0
185.0
192.0
195.0
199.1
205.0
208.3
214.0
217.0
223.0
226.0
228.4
234.3
236.3
240.1
243.1
244.1
246.2
247.2
256.3
258.3
Intensity, cps
m/z, Da

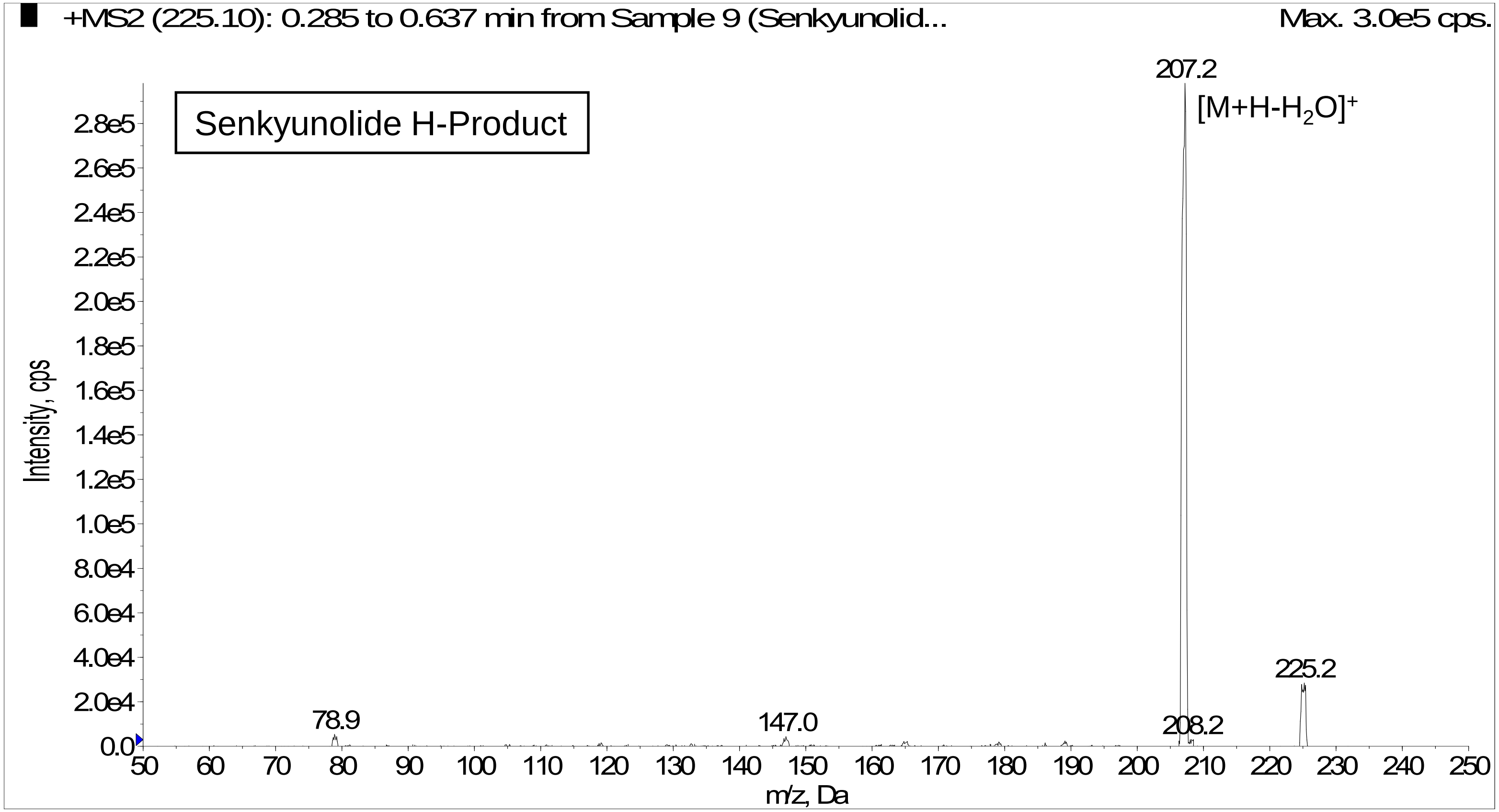

+MS2 (225.10): 0.285 to 0.637 min from Sample 9 (Senkyunolid...
Max. 3.0e5 cps.
Senkyunolide H-Product
207.2
[M+H-H2O]+
225.2
208.2
147.0
78.9
Intensity, cps
m/z, Da

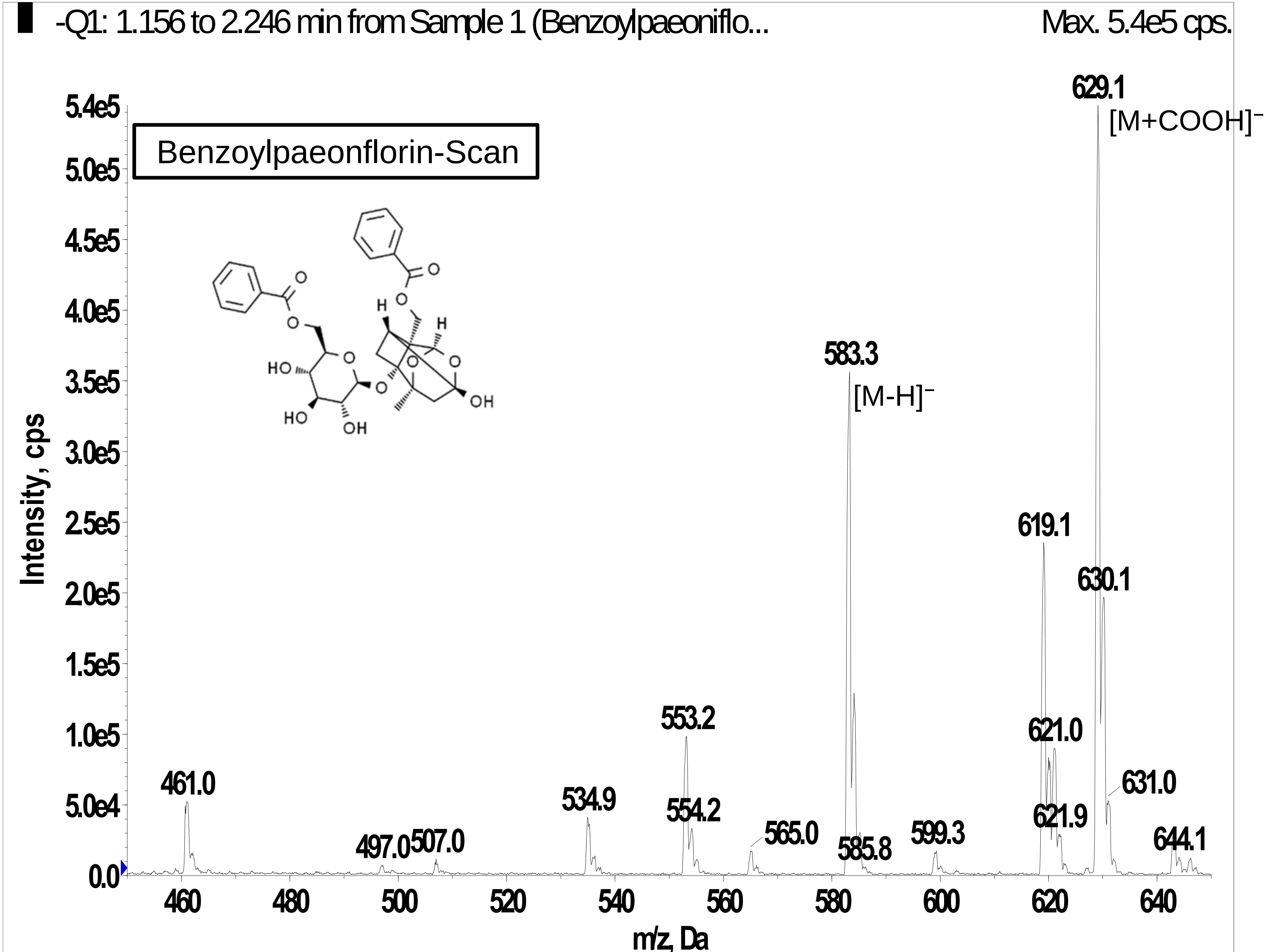

-Q1: 1.156 to 2.246 min from Sample 1 (Benzoylpaeoniflo...
Max. 5.4e5 cps.
Benzoylpaeonflorin-Scan
629.1
[M+COOH]⁻
583.3
[M-H]⁻
619.1
630.1
553.2
621.0
461.0
631.0
534.9
554.2
621.9
565.0
599.3
497.0
507.0
585.8
644.1
Intensity, cps
m/z, Da
HO
OH
O
H

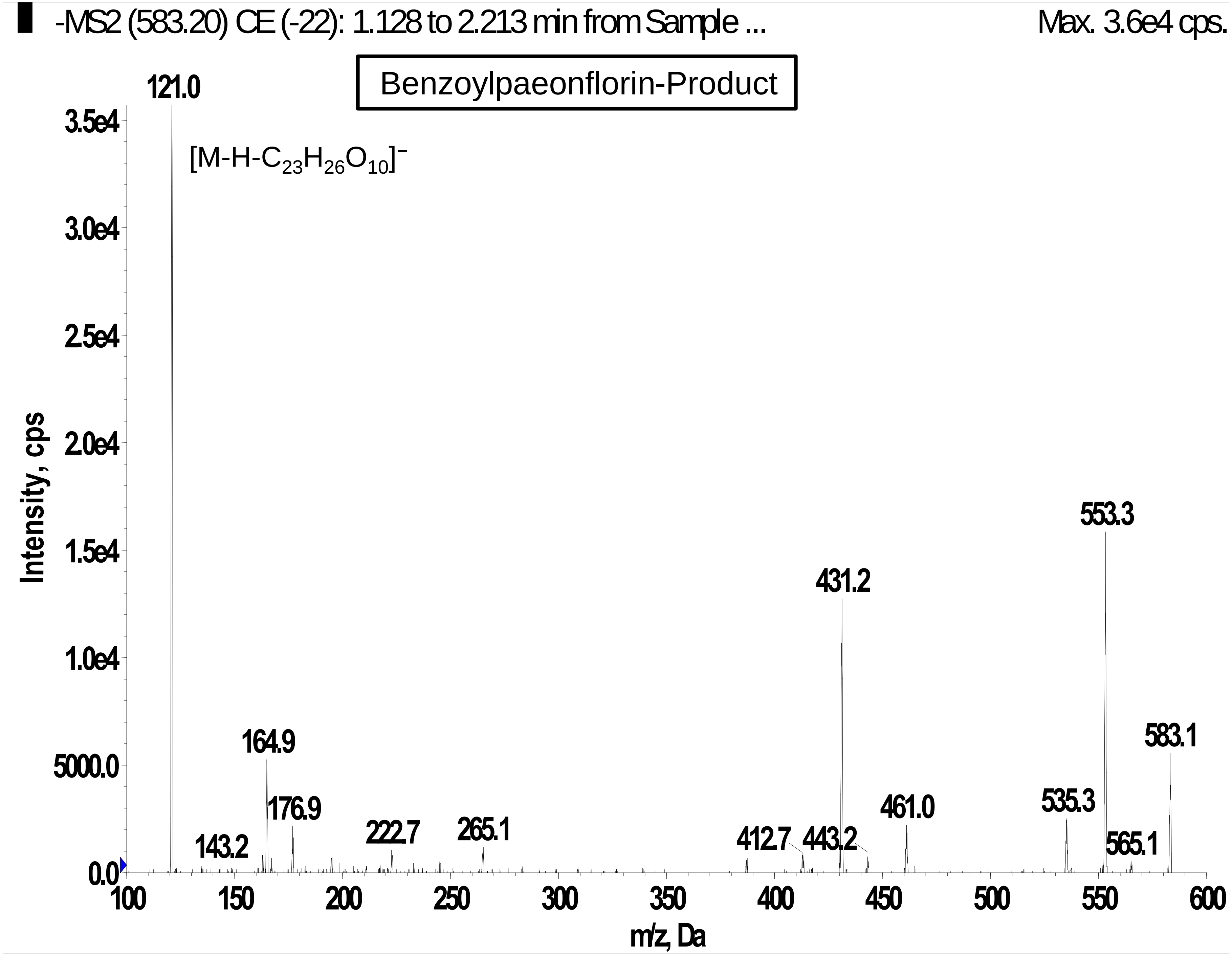

-MS2 (583.20) CE (-22): 1.128 to 2.213 min from Sample ...
Max. 3.6e4 cps.
Benzoylpaeonflorin-Product
[M-H-C23H26O10]−
121.0
164.9
143.2
176.9
222.7
265.1
412.7
443.2
431.2
461.0
535.3
553.3
565.1
583.1
Intensity, cps
3.5e4
3.0e4
2.5e4
2.0e4
1.5e4
1.0e4
5000.0
0.0
100
150
200
250
300
350
400
450
500
550
600
m/z, Da

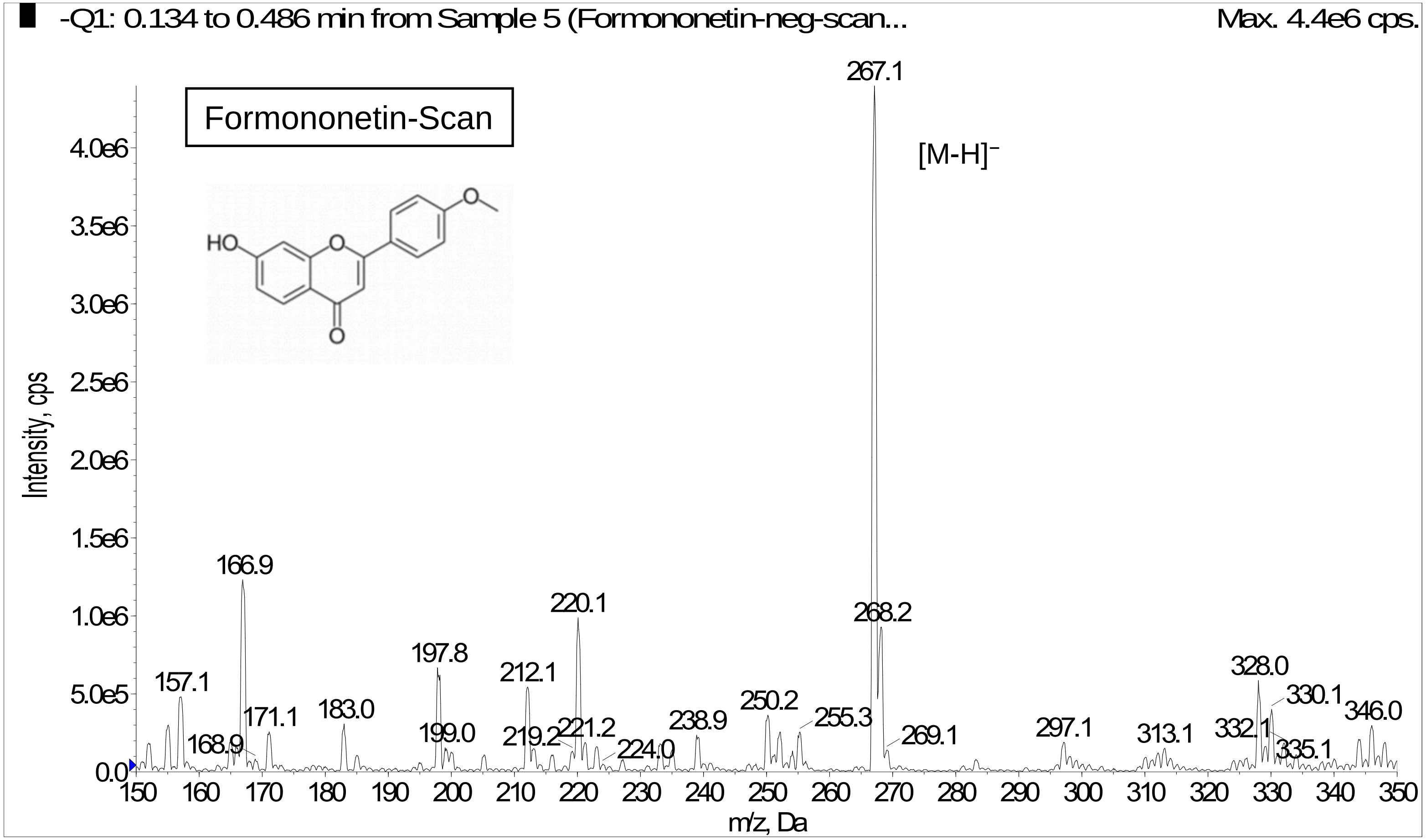

-Q1: 0.134 to 0.486 min from Sample 5 (Formononetin-neg-scan...
Max. 4.4e6 cps.
Formononetin-Scan
HO
O
O
O
267.1
[M-H]⁻
166.9
220.1
268.2
197.8
212.1
157.1
328.0
330.1
183.0
171.1
250.2
255.3
346.0
238.9
221.2
297.1
168.9
199.0
219.2
269.1
313.1
332.1
224.0
335.1
Intensity, cps
m/z, Da

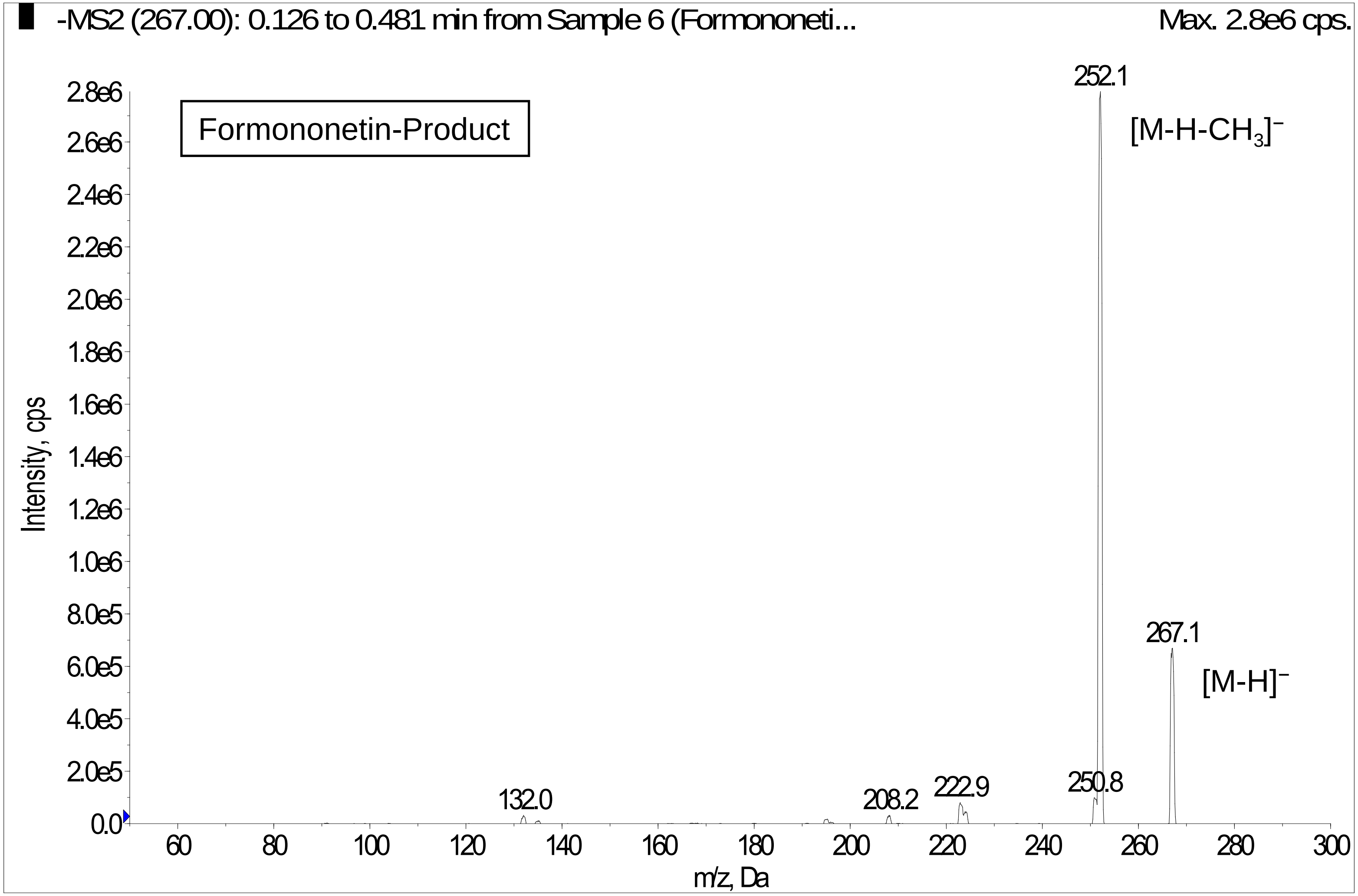

-MS2 (267.00): 0.126 to 0.481 min from Sample 6 (Formononeti...
Max. 2.8e6 cps.
Formononetin-Product
252.1
[M-H-CH3]⁻
267.1
[M-H]⁻
132.0
208.2
222.9
250.8
Intensity, cps
m/z, Da
2.8e6
2.6e6
2.4e6
2.2e6
2.0e6
1.8e6
1.6e6
1.4e6
1.2e6
1.0e6
8.0e5
6.0e5
4.0e5
2.0e5
0.0
60
80
100
120
140
160
180
200
220
240
260
280
300

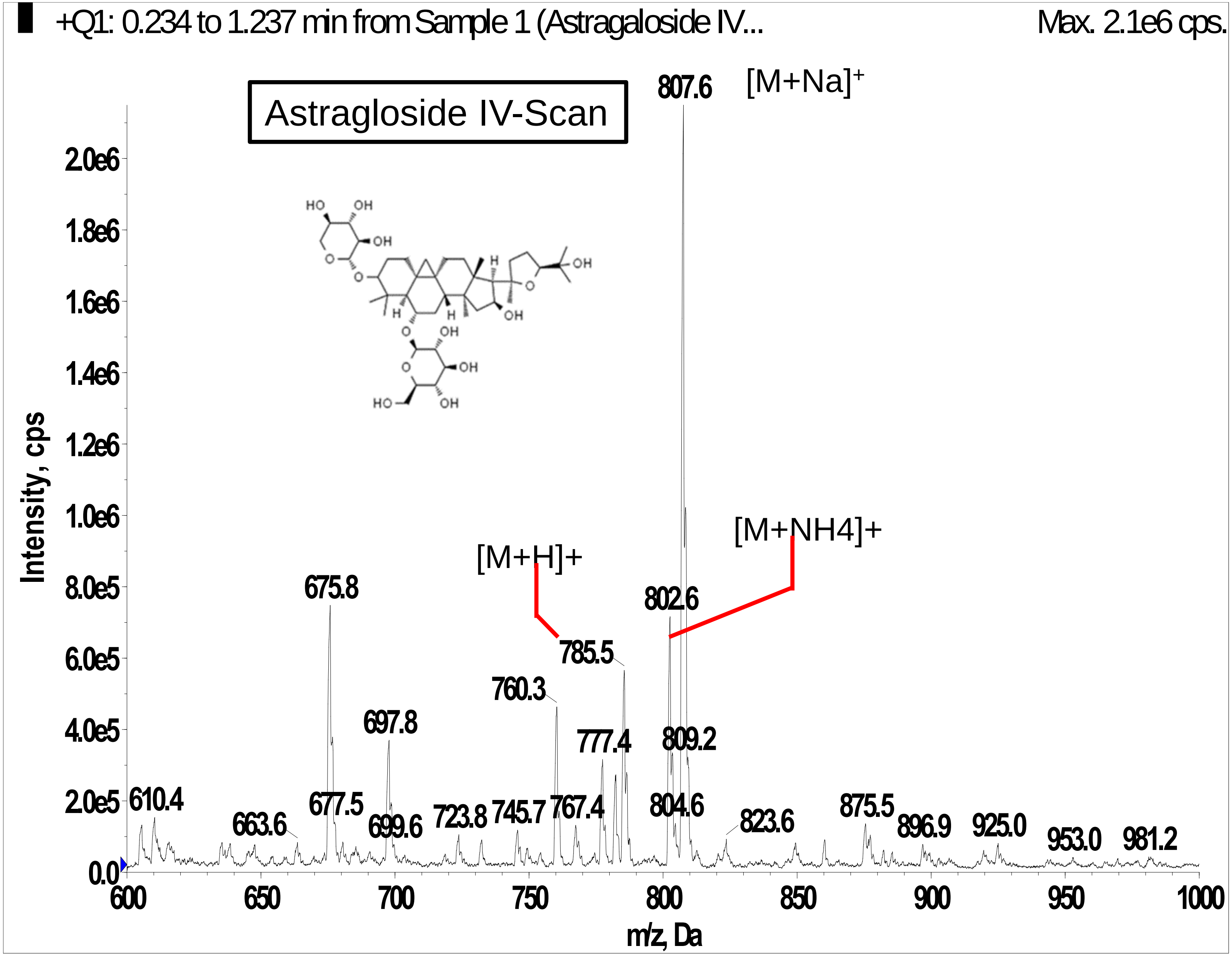
+Q1: 0.234 to 1.237 min from Sample 1 (Astragaloside IV...
Max. 2.1e6 cps.
Astragloside IV-Scan
807.6
[M+Na]+
[M+NH4]+
[M+H]+
675.8
802.6
785.5
760.3
697.8
777.4
809.2
610.4
663.6
677.5
699.6
723.8
745.7
767.4
804.6
823.6
875.5
896.9
925.0
953.0
981.2
Intensity, cps
m/z, Da
2.0e6
1.8e6
1.6e6
1.4e6
1.2e6
1.0e6
8.0e5
6.0e5
4.0e5
2.0e5
0.0
600
650
700
750
800
850
900
950
1000

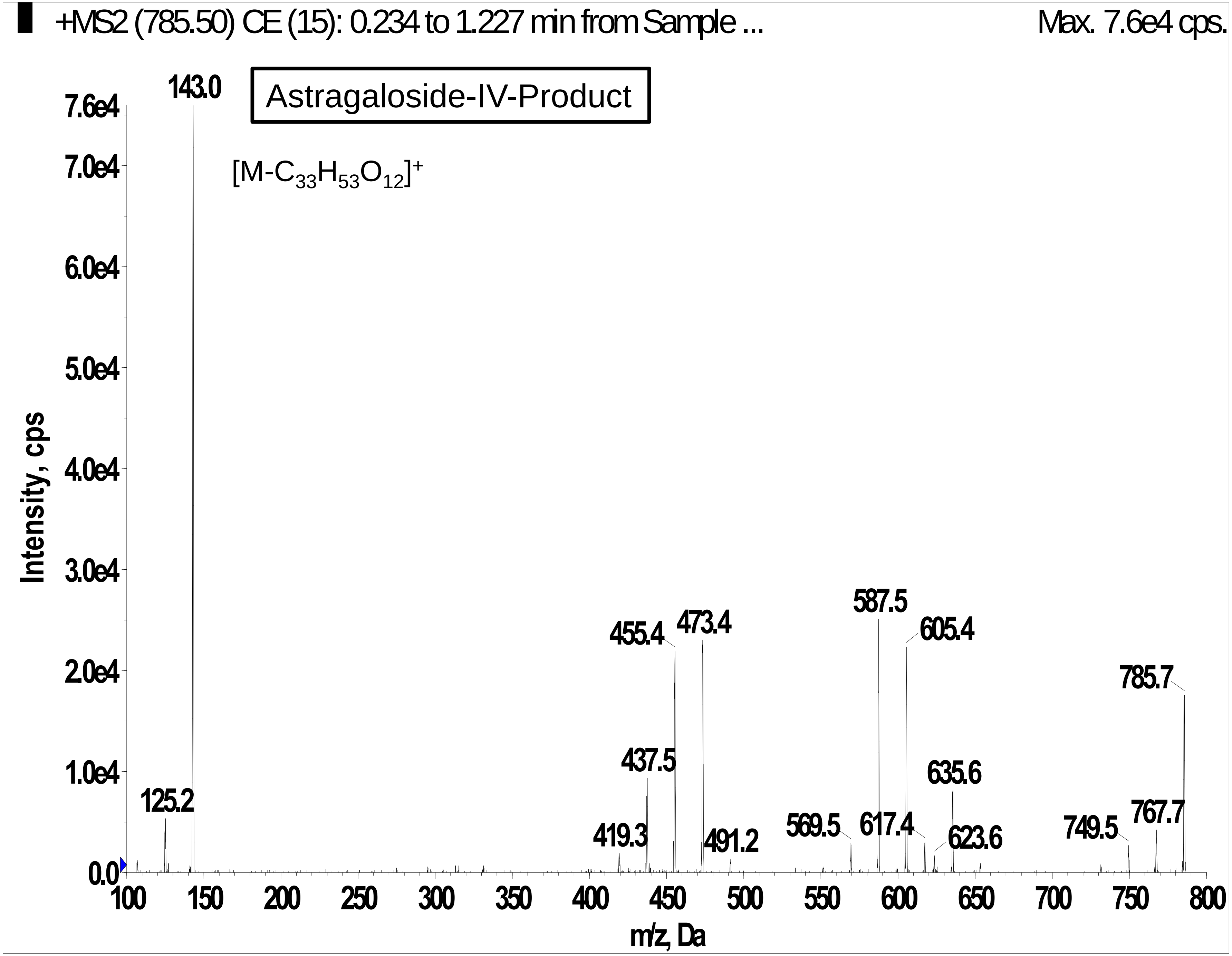
+MS2 (785.50) CE (15): 0.234 to 1.227 min from Sample ...
Max. 7.6e4 cps.
Astragaloside-IV-Product
[M-C33H53O12]+
Intensity, cps
m/z, Da
143.0
125.2
419.3
437.5
455.4
473.4
491.2
569.5
587.5
605.4
617.4
623.6
635.6
749.5
767.7
785.7
