# Supplementary material for: BYHW Decoction Improves Cognitive Impairments in Rats with Cerebral Microinfarcts via Activation of the PKA/CREB Pathway
Source: Oxid Med Cell Longev. 2022 Dec 30;2022:4455654. doi: 10.1155/2022/4455654 (PMC9822752; doi:10.1155/2022/4455654)
Supplement: Supplementary Materials — Graphical abstract. In this study, we applied microsphere-induced cerebral microinfarct (CMI) model in rats to investigate the behavioral and molecular consequences of CMIs. We used the Morris water maze, quantitative proteomics, and other molecular assays and found that activation of the PKA/CREB pathway by BYHW decoction treatment may reverse mitochondrial dysfunction, regulate the generation of ROS and ATP, inhibit apoptosis of hippocampal neurons, and ameliorate CMI-induced cognitive impairments in rats. Collectively, these findings confirmed the therapeutic potential of the BYHW decoction in treating cognitive impairments induced by CMIs and demonstrated a viable mechanism for its action. Supplementary Figure 1: original image of MS1 and MS2 in the chromatograms of the main identified components of BYHW decoction, including Amygdalin, Hydroxysaffor yellow A, Paeoniflorin, Ferulic acid, Senkyunolide I, Senkyunolide H, Benzoylpaeoniflorin, Formononetin, and Astragaloside IV. Supplementary Table 1: a gradient eluting program in UPLC (A) and parameters in MS (B). Supplementary Figure 2: ROS production was measured in primary hippocampal neurons with MitoSOX Red staining, with Hoechst 33342 to label all cell nuclei. Representative images are shown (magnification, 20×), image in each inset. The quantified levels of ROS are expressed as the means ± SD. n = 5. ∗P < 0.05 and ∗∗∗P < 0.001 compared with the OGD group. [file 4455654.f1.zip › Supplementary Table 1 editable.docx]

TABLE 1 A gradient eluting program in UPLC (A) and parameters in MS (B).

| A |  |  |  |  |
| --- | --- | --- | --- | --- |
|  | Time(min) | Flow Rate | %A | %B |
|  | Initial | 0.2 | 10 | 90 |
|  | 4.0 | 0.2 | 20 | 80 |
|  | 9.0 | 0.2 | 60 | 40 |
|  | 12.0 | 0.2 | 100 | 0 |
|  | 14.0 | 0.2 | 100 | 0 |
|  | 14.1 | 0.2 | 10 | 90 |
|  | 18.0 | 0.2 | 10 | 90 |

B

| Compounds | Q1 | Q3 | Retention Time(min) | DP | CE |
| --- | --- | --- | --- | --- | --- |
| Ferulic acid | 193.0 | 134.0 | 9.74 | -70 | -20 |
| Paeoniflorin | 479.2 | 121.1 | 9.15 | -130 | -35 |
| Hydroxysafflor yellow A | 611.2 | 491.2 | 9.02 | -80 | -38 |
| Formononetin | 267.0 | 252.1 | 12 | -110 | -30 |
| Amygdalin | 456.1 | 322.8 | 7.03 | -150 | -20 |
| Benzoylpaeoniflorin | 583.3 | 121.0 | 11.2 | -145 | -35 |
| Astragaloside IV | 785.5 | 143.0 | 13.1 | 120 | 21 |
| Senkyunolide I | 225.2 | 207.3 | 10.7 | 65 | 10 |
| Senkyunolide H | 225.1 | 207.2 | 10.9 | 65 | 23 |
